# SAF-A Regulates Interphase Chromosome Structure through Oligomerization with Chromatin-Associated RNAs

**DOI:** 10.1016/j.cell.2017.05.029

**Published:** 2017-06-15

**Authors:** Ryu-Suke Nozawa, Lora Boteva, Dinesh C. Soares, Catherine Naughton, Alison R. Dun, Adam Buckle, Bernard Ramsahoye, Peter C. Bruton, Rebecca S. Saleeb, Maria Arnedo, Bill Hill, Rory R. Duncan, Sutherland K. Maciver, Nick Gilbert

**Affiliations:** 1 MRC Human Genetics Unit, Institute of Genetics and Molecular Medicine, University of Edinburgh, Crewe Road, Edinburgh EH4 2XU, UK; 2 Centre for Genomics and Experimental Medicine, Institute of Genetics and Molecular Medicine, University of Edinburgh, Crewe Road, Edinburgh EH4 2XU, UK; 3 Centre for Integrative Physiology, Edinburgh Medical School, University of Edinburgh, George Square, Edinburgh EH8 9XD, UK; 4 Edinburgh Super-Resolution Imaging Consortium, Institute of Biological Chemistry, Biophysics, and Bioengineering, Heriot-Watt University, Edinburgh EH14 4AS, UK

**Keywords:** chromatin, transcription, nuclear architecture, chromatin compaction, chromatin-associated RNAs, hnRNPU, SAF-A, AAA^+^ ATPases, chromosome stability

## Abstract

Higher eukaryotic chromosomes are organized into topologically constrained functional domains; however, the molecular mechanisms required to sustain these complex interphase chromatin structures are unknown. A stable matrix underpinning nuclear organization was hypothesized, but the idea was abandoned as more dynamic models of chromatin behavior became prevalent. Here, we report that scaffold attachment factor A (SAF-A), originally identified as a structural nuclear protein, interacts with chromatin-associated RNAs (caRNAs) via its RGG domain to regulate human interphase chromatin structures in a transcription-dependent manner. Mechanistically, this is dependent on SAF-A’s AAA^+^ ATPase domain, which mediates cycles of protein oligomerization with caRNAs, in response to ATP binding and hydrolysis. SAF-A oligomerization decompacts large-scale chromatin structure while SAF-A loss or monomerization promotes aberrant chromosome folding and accumulation of genome damage. Our results show that SAF-A and caRNAs form a dynamic, transcriptionally responsive chromatin mesh that organizes large-scale chromosome structures and protects the genome from instability.

## Highlights

• SAF-A remodels interphase chromosome structure
• Transcription regulates SAF-A oligomerization with caRNAs
• SAF-A and caRNAs interact to form a chromatin mesh
• Chromatin structure affects genome stability

## Graphical Abstract

**Figure:**
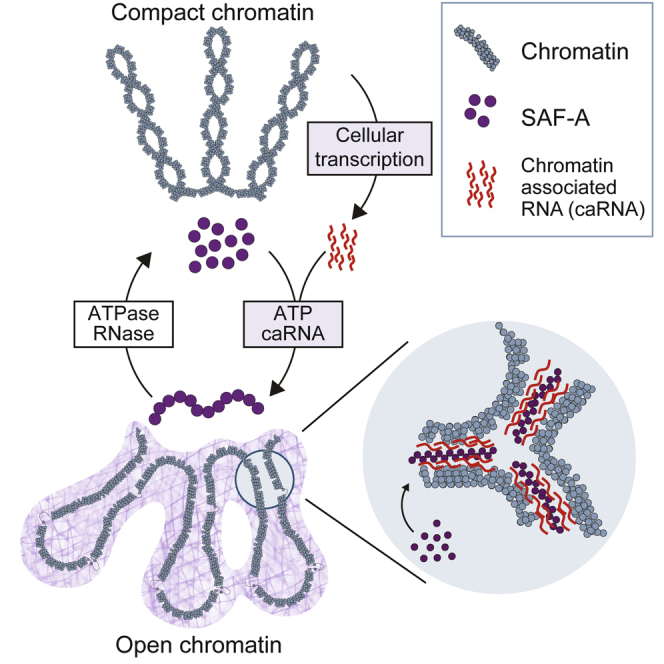

## Teaser

A scaffolding protein interacts with chromatin-associated RNAs to regulate human interphase chromatin structures in a transcription-dependent manner.

## Introduction

Mammalian interphase chromosomes are organized into topologically constrained chromatin domains (Belmont et al., 1989), which are responsive to transcription (Naughton et al., 2013) and local gene density (Goetze et al., 2007). Gene-poor genomic regions have a compact large-scale chromatin structure, while regions rich in genes and transcriptional activity have a more decompacted structure (Gilbert et al., 2004, Goetze et al., 2007, Naughton et al., 2010). Previously, we suggested that transcription and topoisomerase activities, that occur at the gene level, alter local topology to form supercoiling domains (Naughton et al., 2013) and these correspond to structures seen by Hi-C (Rao et al., 2014). It is unclear, however, how these processes could impact on large-scale chromatin structures.

In contrast to mitosis, where topoisomerases and condensin play a central role in scaffolding chromatin (Samejima et al., 2012), the molecular underpinnings of interphase domains are poorly characterized (Belmont, 2014) and their functional impact remains unknown. A “nuclear matrix” consisting of insoluble proteins and RNA particles was proposed to organize interphase chromatin architecture (Berezney and Coffey, 1974, Pederson, 1998), maintain chromosome territories (Ma et al., 1999), enhance gene expression, and provide a platform for nuclear processes, but chromatin mobility in vivo (Chubb et al., 2002, Phair et al., 2004) and the lack of a stable nucleoprotein structure in live cells undermined the concept (Hancock, 2000). However, the structural contribution of RNA to chromatin organization remains undisputed: a large proportion of chromatin by mass corresponds to RNA (Holmes et al., 1972), mostly belonging to the loosely termed chromatin-associated RNA (caRNA) class. The functional roles of caRNAs are hinted by the observation that they are stably associated with interphase chromosome territories (Fey et al., 1986, Hall and Lawrence, 2016) and their disruption leads to chromatin condensation (Hall et al., 2014). The molecular basis for this is unknown, but it is thought heterogeneous ribonucleoprotein particles (hnRNPs) provide a docking platform to associate with nascent transcripts (Melé and Rinn, 2016) while caRNAs might influence chromatin structure (Caudron-Herger and Rippe, 2012). Recent studies have been unable to find specific species of RNA, similar to XIST, which could regulate large-scale chromatin structure, suggesting instead that diverse caRNAs transiently interact with chromatin, forming a dynamic compartment (Melé and Rinn, 2016). However, a refinement of this model would require insight into how the interaction of proteins with caRNAs can regulate chromatin structure.

Scaffold attachment factor A (SAF-A), also known as heterogeneous ribonucleoprotein U (HNRNP-U) (Kiledjian and Dreyfuss, 1992, Romig et al., 1992), is an abundant protein reported to bind scaffold attachment regions (Göhring et al., 1997) and involved in several cellular processes such as pre-mRNA splicing (Xiao et al., 2012), accumulation at DNA damage sites (Britton et al., 2014) and Xist-mediated transcriptional silencing (McHugh et al., 2015). Structurally, SAF-A contains a low complexity RNA-binding RGG repeat and an ATP-binding AAA^+^ domain, known to facilitate the assembly (Erzberger and Berger, 2006) and operation of diverse protein and nucleoprotein machines. Other well-characterized AAA^+^ domain-containing proteins, such as replication factor C (RFC) and DnaA, oligomerize through their AAA domains, often with nucleic acids, to form higher molecular weight structures. We characterized SAF-A activity to understand the relationship between its structure and function in regulating chromatin architecture.

We are able to demonstrate that SAF-A regulates transcriptionally active large-scale chromatin structures in human cells. Using functional mutants of SAF-A, we dissect the underlying molecular mechanisms to show that SAF-A can cycle from a monomeric to a homo-oligomeric state through ATP binding and caRNAs; concomitantly, SAF-A oligomerization drives chromatin decompaction while monomerization compacts large-scale chromatin organization.

We suggest that SAF-A interacts with caRNAs to form a chromatin mesh (Nozawa and Gilbert, 2014), and unlike the historical concept of a nuclear matrix, is highly responsive to ongoing transcription and can undergo dynamic cycles of assembly and disassembly. Surprisingly, a gross change in chromatin structure has limited effect on transcription indicating that gene-level chromatin structure is important for regulating transcription, rather than large-scale chromatin architecture. Although changes in large-scale chromatin structure do not influence gene expression, an alteration in the chromatin landscape had a dramatic effect on genome stability; loss or mutation of SAF-A triggered a DNA damage response and chromosomal instability.

To unify the structural and enzymatic aspects of SAF-A function, we speculate that SAF-A/RNA interactions will drive the formation of local chromatin domains or micro-bodies (Brackley et al., 2016), while its role in regulating large-scale chromatin structures will partition the genome into functionally diverse segments. This process is essential for maintaining genome stability and could provide a constraint to maintain clusters of genes together in the genome during evolution (Ghanbarian and Hurst, 2015).

## Results

### SAF-A Remodels Chromatin Structures

**Figure 1:**
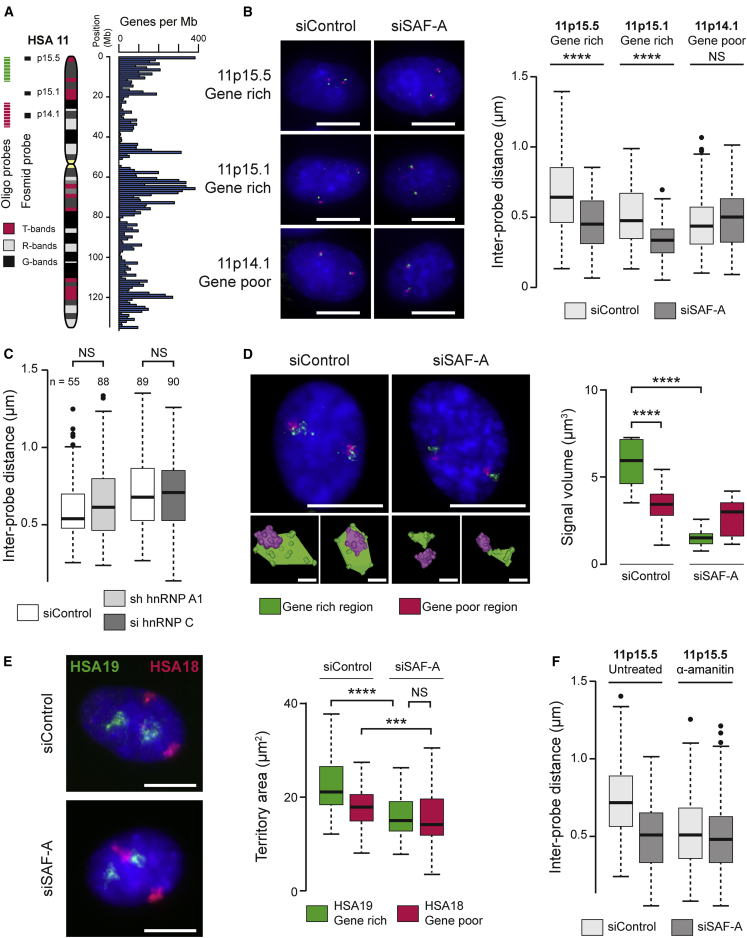
Transcription-Dependent SAF-A Regulation of Interphase Chromosome Structures (A) Ideogram of human chromosome 11 (HSA11) showing locations of fosmid probes at p15.5, p15.1, and p14.1 and oligo probe clusters located in gene-rich (green) and gene-poor (red) regions. (B) FISH assay for large scale chromatin compaction with probes (red and green) at gene-rich 11p15.5, 11p15.1, or gene-poor 11p14.1 loci in human RPE1 cells. Left: Representative images for cells (siControl) or cells depleted of SAF-A (siSAF-A); nuclei were counterstained with 4′,6-diamidino-2-phenylindole (DAPI; blue). Scale bars, 10 μm. Right: Boxplots showing distribution of distances between probe pairs. Shaded boxes show median and interquartile range of data (n = 70 nuclei for at least two biological replicates). (C) Boxplots of distances between probe pairs at 11p15.5 from a FISH assay in cells (siControl) or cells depleted of hnRNP A1 or hnRNP C. (D) Left: FISH with fluorescently labeled oligo probes covering gene-rich (see A; green) or gene-poor region (see A; red) in cells (siControl) or cells depleted of SAF-A (siSAF-A). Scale bars, 10 μm. Inset: 3D reconstructions of probe clusters to facilitate visualization. Scale bars, 1 μm. Right: Quantification of volumes occupied by probe clusters (n > 20 nuclei for each sample). (E) Left: FISH for chromosome 18 (HSA18) and 19 (HSA19) territories in cells (siControl) or cells depleted of SAF-A (siSAF-A). Scale bars, 10 μm. Right: quantification of territory area (n > 50 nuclei for each sample). (F) Boxplots showing the distribution of distances between probe pairs at 11p15.5 from a FISH assay in cells (siControl) or cells depleted of SAF-A (siSAF-A) in the presence and absence of the transcription inhibitor α-amanitin (5 hr). All conditions are significantly different from the untreated control (p < 0.0001, n = 100 nuclei for at least two biological replicates). p values for a Wilcoxon test: NS, not significant; ^∗∗∗^p < 0.001; ^∗∗∗∗^p < 0.0001. See also Figure S1 and Table S1.

**Figure S1:**
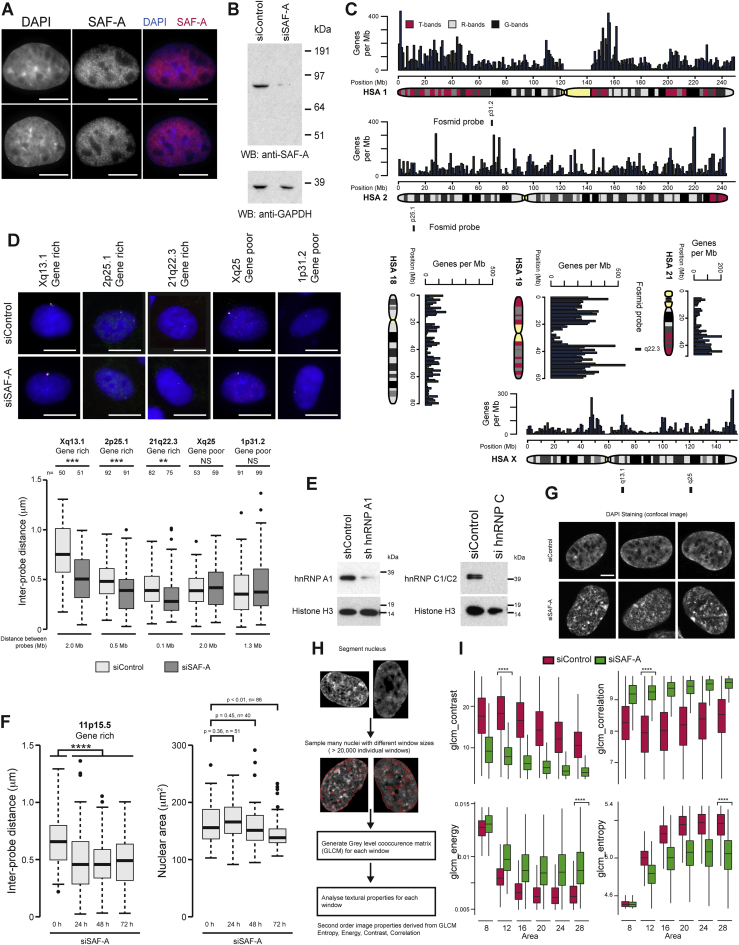
Characterization of SAF-A’s Ability to Regulate Interphase Chromosome Structures, Related to Figure 1 (A) Immunofluorescence staining showing the distribution of SAF-A (red) and DAPI-stained DNA (blue) in ARPE-19 cells. Scale bars, 10 μm. (B) western blot for SAF-A and GAPDH in RPE1 cells (siControl) and cells depleted of SAF-A (siSAF-A). (C) Ideogram of human chromosome 1 (HSA1), 2 (HSA2), 18 (HSA18), 19 (HSA19), 21 (HSA21) and X (HSA X) showing fosmid probe pairs used in this study at 1p31.2, 2p25.1, 21q22.3, Xq13.1 and Xq25. Whole chromosome paints were used for HSA 18 and HSA 19. (D) Top, representative images of FISH experiments showing control cells (siControl) or cells depleted of SAF-A (siSAF-A). Scale bars, 10 μm. Bottom, boxplots of inter-probe distances at gene rich Xq13.1, 2p25.1 and 21q22.3 regions and gene poor Xq25 and 1p31.2 from a FISH chromatin compaction assay in RPE1 cells. P values for a Wilcoxon test (n > 50; NS = not significant; ^∗∗^p < 0.01; ^∗∗∗^p < 0.001). (E) western blot for hnRNPA1 (left panel) or hnRNPC (right panel) and histone H3 in RPE1 control cells (siControl) and cells depleted of hnRNP A1 (left) or hnRNP C (right). (F) Left, boxplots of distances between probe pairs at 11p15.5 from a FISH assay in control cells (siControl) or cells depleted of SAF-A (siSAF-A) for increasing amount of time (24, 48, 72 h). Right, boxplots of nuclear area in control cells (0 h) or cells depleted of SAF-A (siSAF-A) for increasing times (24, 48, 72 h). (G) Confocal images of DAPI-stained nuclei in control RPE1 cells (siControl) and RPE1 cells depleted of SAF-A (siSAF-A). Scale bar, 5 μm. (H) Diagram showing how nuclei are sampled to measure DNA texture. (I) Boxplots for different sample window sixes (x axis) for different texture properties (y axis) in control (red) and SAF-A depleted (green) cells. Contrast – local intensity variation; Correlation – gray level linear dependence; Energy – homogeneity (a homogeneous scene will have only a few gray levels); Entropy – homogeneity (Homogeneous scenes have a high entropy).

SAF-A’s contribution to chromatin structure is unknown; it is nuclear diffuse (Figure S1A), but excluded from condensed DAPI bright regions such as the gene-poor nuclear periphery. SAF-A’s abundance, localization, and structural characteristics suggest it may play a role in organizing nuclear architecture (Hall and Lawrence, 2016). To examine whether SAF-A affects large-scale chromatin, we analyzed chromatin compaction using three-dimensional (3D) DNA fluorescence in situ hybridization (FISH) across loci with different epigenetic states and distinct levels of chromatin folding (Boettiger et al., 2016, Gilbert et al., 2004) using fosmid probe pairs (Figures 1A and S1C; Table S1) (detailed in Naughton et al., 2013). SAF-A small interfering RNA (siRNA) treatment resulted in a substantial reduction of SAF-A protein (Figure S1B) and caused a significant compaction of gene-rich 11p15.5 and 11p15.1 loci enriched in “open” chromatin, but not the gene-poor 11p14.1 locus (Figure 1B), indicating that SAF-A is involved in decompacting chromatin. Similar changes in chromatin structure were observed for additional gene-rich (2p25.1, 21q22.3, Xq13.1) and gene-poor loci (Xq25, 1p31.2) using fosmid probes positioned 0.1–2.0 Mb apart (Figure S1D). Depletion of other hnRNP members, such as HNRNP A1 and HNRNP C, showed no obvious nuclear effects (Figure 1C), indicating that SAF-A has a unique role among hnRNPs in regulating chromatin structure. To extend our analysis, we targeted labeled oligo probes to gene-rich and gene-poor regions on chromosome 11 (Figure 1A) and analyzed chromatin compaction by FISH and 3D modeling. SAF-A depletion led to significant compaction of the HSA11 gene-rich domain, but not the gene-poor region (Figure 1D), indicating that SAF-A contributes to the partitioning of the genome into segments with distinct structures (Boettiger et al., 2016). Changes in chromatin compaction following SAF-A depletion were observed by 24 hr, while global changes in nuclear area were not apparent until later, indicating that the effect is due to changes in local chromatin structure rather than an alteration in nuclear size (Figure S1F).

The human genome is partitioned into chromosomes with very different gene densities; we reasoned that if SAF-A predominantly regulated gene-rich chromosome regions, its depletion would have a greater effect on gene-rich, rather than gene-poor chromosomes. To test this, we monitored the chromosome territory of the gene-rich HSA19 and gene-poor HSA18 before and after SAF-A depletion. The two similarly sized chromosomes have different gene densities (45 genes/Mb on HSA18 versus 216 genes/Mb on HSA19) (Figure S1C), and HSA19 has been shown to adopt a more open chromosome territory configuration than HSA18 (Croft et al., 1999). Upon SAF-A depletion, we observed significant chromosome territory compaction, but HSA19 was compacted significantly more that HSA18, consistent with SAF-A predominantly regulating gene-rich chromosome regions at scales up from 100 kb to a whole chromosome territory (Figure 1E). At the largest nuclear scale, we also observed a significant change in DAPI texture (Figures S1G and S1H) after SAF-A depletion.

Previously, we reported that transcription also decompacts large scale chromosome structures (Naughton et al., 2010). To determine whether SAF-A and transcription act independently or in a synergistic manner, we analyzed chromatin compaction in control cells or cells depleted of SAF-A following treatment with the transcription inhibitor α-amanitin. Inhibition of transcription and loss of SAF-A together did not result in a further compaction of chromatin than either of the two factors alone, consistent with the two processes functioning together (Figure 1F).

### Transcriptional Upstream Regulation of SAF-A

**Figure 2:**
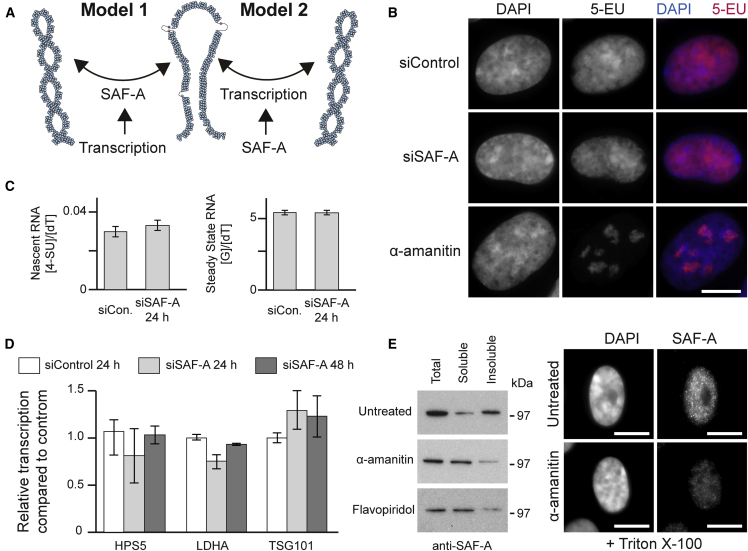
SAF-A Modulation of Chromatin Structure Is Downstream and Regulated by Gene Transcription (A) Alternate models depicting the relationship between transcription, SAF-A, and chromatin structure. (B) Fluorescence microscopy of cells pulse labeled (30 min) with 5-EU, fixed and conjugated, using click chemistry with Cy5 azide. Control cells (siControl), cells depleted of SAF-A (siSAF-A), or treated with α-amanitin (5 h). Scale bar, 10 μm. (C) Bar graph showing nascent and steady-state RNA in control RPE1 cells or cells depleted of SAF-A quantified by measuring the pulsed incorporation of 4-SU, guanine, and thymidine. Error bars represent SD for two biological replicates. (D) Relative expression of HPS5, LDHA, and TSG101 in control cells and cells depleted of SAF-A measured by qRT-PCR. Error bars are SEM for at least two biological replicates. (E) Left: Western blot showing fractionation of SAF-A protein into soluble and insoluble fractions in control cells or cells treated (5 hr) with α-amanitin or flavopiridol. Right: Immunofluorescence for SAF-A in Triton X-100 extracted cells treated with α-amanitin and counterstained with DAPI. Scale bars, 10 μm. See also Figure S2.

**Figure S2:**
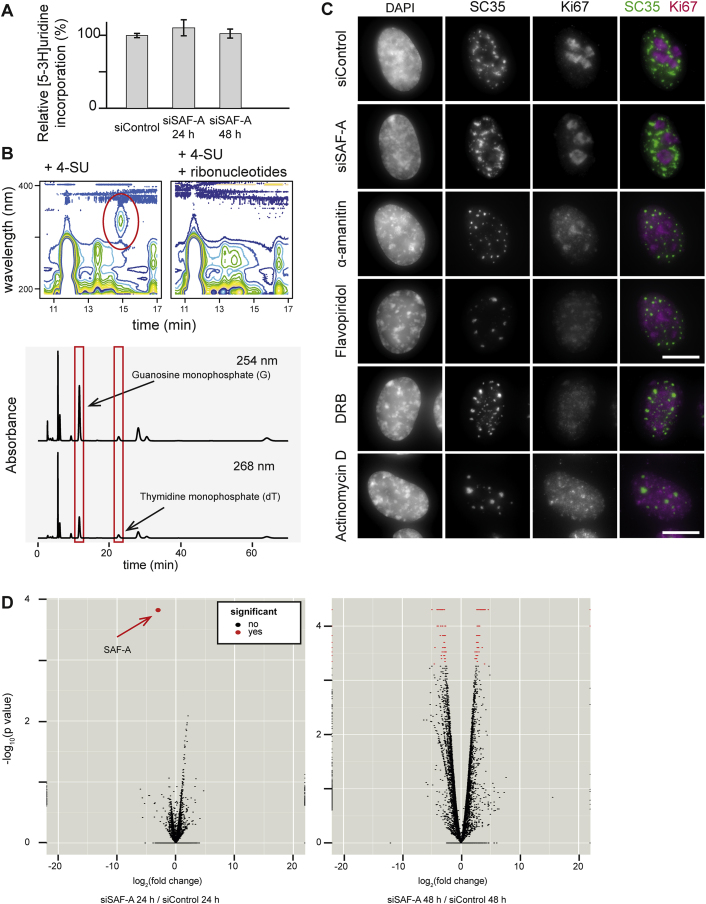
SAF-A Loss Does Not Impact on Gene Transcription, Related to Figure 2 (A) Bar graph showing [5-3H]uridine incorporation into nascent RNA in control RPE1 cells or cells depleted of SAF-A. Error bars are SEM for at least two biological replicates. (B) HPLC traces showing the identification and quantification of 4-SU nucleoside monophosphate (top, red circle), guanine monophosphate and thymidine mono phosphate peaks (bottom, red boxes) after isolating and fractionating nucleosides from whole cell extracts pulsed with 4-SU in the presence or absence of competitor ribonucleosides. (C) Immunofluorescence staining for Ki67 (red) or SC35 (green) in RPE1 control cells (siControl), cells depleted for SAF-A (siSAF-A) or treated with transcription inhibitor α-amanitin, flavopiridol, actinomycin D or DRB (each for 5 h). Scale bar, 10 μm. (D) Volcano plots for RNA-seq data for RPE1 control cells compared to cells depleted of SAF-A for 24 or 48 hr. Significantly differently expressed genes (q < 0.05) are shown in red.

We reasoned that if SAF-A and transcription acted together, there could be two potential models for the functional relationship between SAF-A, transcription, and chromatin: either transcription could regulate SAF-A to alter chromatin structure (model 1) or SAF-A could have an impact on transcription, which regulates structure (model 2) (Figure 2A). To discriminate between these possibilities, we investigated the effect of SAF-A depletion on cellular transcription using different approaches. First, we examined global transcription via a short pulse of 5-ethynyl uridine (5-EU) incorporated into newly transcribed RNA (∼80% pol II transcripts) (Jackson et al., 2000) and quantified by fluorescent labeling (Figure 2B). Depletion of SAF-A had little effect on the distribution of transcripts or the apparent levels of transcription. In contrast, inhibition of transcription using α-amanitin had a profound effect on newly synthesized RNA. To confirm these results, [5-^3^H]uridine was pulsed into cells and measured in newly synthesized RNA; there was no change in transcription after 24 or 48 hr of SAF-A depletion (Figure S2A). This analysis was extended by pulsing cells with 4-thiouridine before analyzing the nucleoside composition of RNA and DNA (Figure S2B). SAF-A depletion had little effect on either nascent RNA or steady-state RNA levels (Figure 2C). Consistently, cytological markers of transcription (Ki67 and SC35) showed no changes in their distribution after SAF-A depletion (Figure S2C). To determine if individual transcripts were affected, we analyzed mRNA by next generation sequencing. At 24 hr following depletion, only SAF-A was differentially expressed compared to control cells, while at 48 hr, there were limited changes in cell-cycle-dependent genes (Figure S2D) consistent with other studies (Xiao et al., 2012, Ye et al., 2015). These results were validated by qRT-PCR (Figure 2D) providing overwhelming evidence that transcription is upstream of SAF-A (model 1). To further support model 1, we reasoned that changes in transcription might be expected to influence SAF-A behavior. In control cells SAF-A is insoluble (Figure 2E), but after transcription, inhibition SAF-A is released into a soluble fraction consistent with transcription being functionally upstream of SAF-A (model 1). These surprising results indicate that while SAF-A exerts a significant effect on large-scale chromatin structures (Figure 1), there is a limited concomitant effect on transcription, suggesting it is small-scale chromatin structures that predominantly impact transcriptional processes.

### SAF-A Actuation by ATP Binding

**Figure 3:**
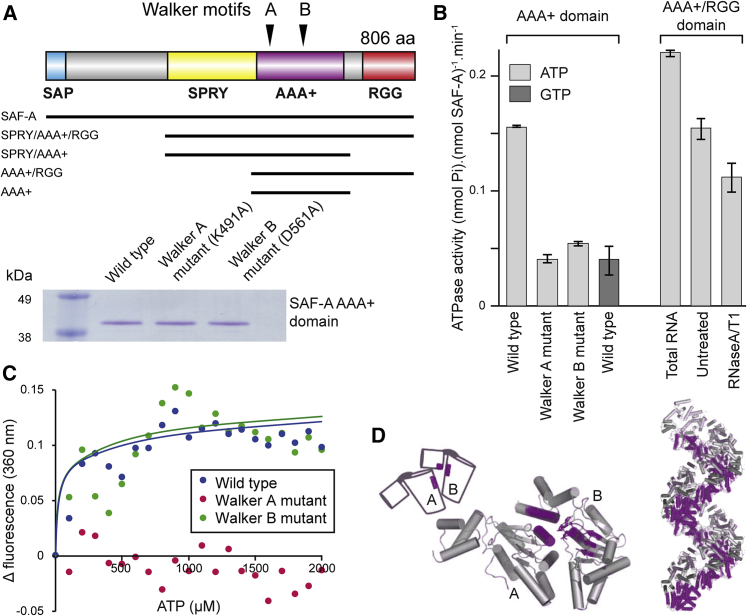
SAF-A Possesses an RNA-Dependent ATPase Activity and Is Actuated by ATP Binding (A) Top: Diagram detailing SAF-A domains (SAP, SPRY, AAA^+^, and RGG), Walker A/B motifs, and constructs used in this study. Walker A mutation was K491A while Walker B mutation was D561A. Bottom: Coomassie-stained SDS-PAGE of purified SAF-A AAA^+^ protein. (B) Left: ATPase or GTPase activity of recombinant wild-type SAF-A AAA^+^ domain and Walker A (deficient in ATP binding) or Walker B (deficient in ATP hydrolysis) SAF-A AAA^+^ mutants. Right: ATPase activity of SAF-A AAA^+^/RGG protein fragment with added total RNA or following RNase treatment. Error bars are SEM for three technical replicates; data is representative of two biological experiments. (C) Fluorescence spectroscopy (Ex 290 nm/Em 360 nm) of SAF-A AAA^+^ protein (blue) or Walker A (red) or Walker B (green) mutants with increasing concentrations of ATP. Data is representative of two biological replicates. (D) Cartoon of ATP-DnaA complex showing interactions between neighboring AAA^+^ modules (purple) to form a helical filament. Image modified from (Erzberger and Berger, 2006). See also Figure S3.

**Figure S3:**
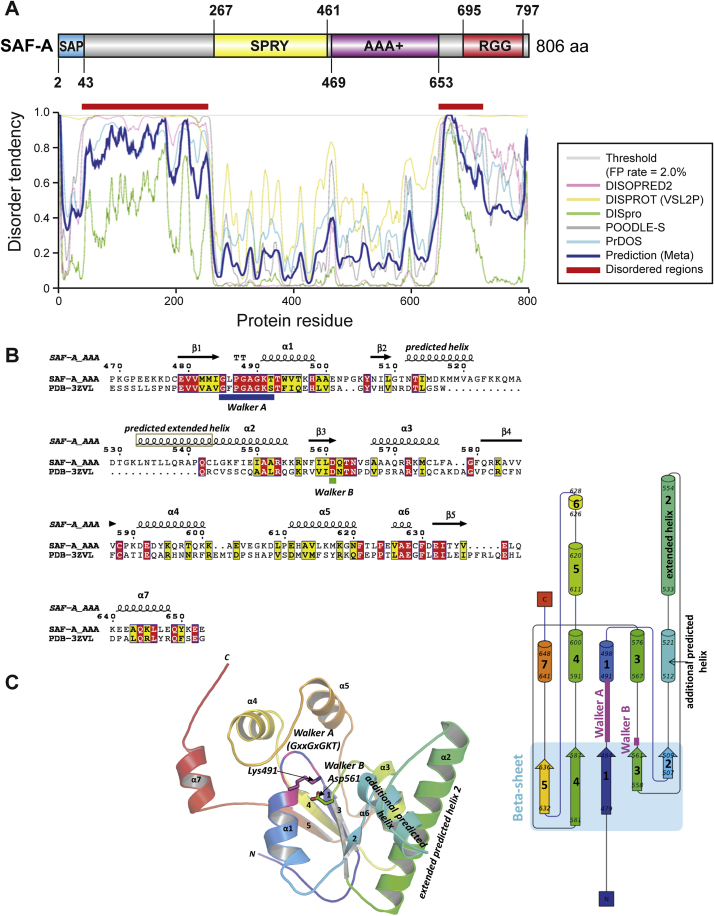
Structural Characteristics of SAF-A and Identification of Walker and Initiator-Specific Motifs, Related to Figure 3 (A) SAF-A encodes four conserved domains: SAP (SAF-A/B, Acinus and PIAS) (Aravind and Koonin, 2000) which has DNA binding activity (Göhring et al., 1997), SPRY (Spla and Ryanodine receptor) of unknown function (Ponting et al., 1997), AAA+ (ATPases Associated with diverse cellular Activities) (Erzberger and Berger, 2006), and a low complexity RGG (arginine glycine-glycine) RNA-binding domain (Helbig and Fackelmayer, 2003, Thandapani et al., 2013). Probability of protein disorder across SAF-A calculated using different algorithms. Amino acid number is given on x axis while y axis depicts the levels of protein disorder. (B) Sequence and structure homology between SAF-A AAA+ domain and mammalian PNK (PDB-3ZVL) structure showing predicted α helices, β sheets and putative Walker motifs. Conserved amino acids are labeled in red and similar amino acids are marked in yellow. (C) Left, predicted ribbon diagram of SAF-A, modeled on PDB-3ZVL, showing putative Walker motifs and ISM. Right, cartoon of SAF-A protein showing key domains and ISM.

To understand the relationship between SAF-A and transcription and how SAF-A mechanistically impacts chromatin, we undertook a structural analysis of the SAF-A protein. SAF-A encodes four conserved domains (Figure 3A): SAP (SAF-A/B, acinus, and PIAS) that has DNA binding activity (Göhring et al., 1997), SPRY (SPla and the ryanodine receptor) of unknown function, AAA^+^ (ATPases associated with diverse cellular activities) (Erzberger and Berger, 2006), and a low complexity RGG (arginine glycine-glycine) RNA-binding domain (Helbig and Fackelmayer, 2003). Globular domain composition and disorder prediction indicate that the SPRY and AAA^+^ domain are ordered but separated from the SAP and RGG domains (Figure S3A), suggesting the SPRY and AAA^+^ domains adopt a defined conformation. 3D homology modeling of SAF-A AAA^+^ domain (Figures S3B and S3C) showed the AAA^+^ domain has a core αβα nucleotide-binding fold containing highly conserved Walker-A (WA), Walker-B (WB), and a helical subdomain known as the “lid.” The Walker A motif is predicted to be necessary for nucleotide binding, while the Walker B motif is required for nucleotide hydrolysis.

We directly tested whether SAF-A AAA^+^ domain could hydrolyze ATP or GTP. Purified SAF-A AAA^+^ fragment, but not SAF-A Walker A or Walker B mutants, exhibited ATPase activity at 0.15 nmol ATP hydrolyzed per nmol of SAF-A AAA^+^ fragment per min, but no GTPase activity (Figure 3B), similar or higher than other AAA^+^ proteins such as DnaA (Sekimizu et al., 1987) and ORC (Klemm et al., 1997) at 0.017 and 0.27 nmol ATP hydrolyzed per nmol protein per min, respectively. As the AAA^+^ domain is located adjacent to an RNA-binding domain, we speculated that RNA might modulate the SAF-A ATPase activity. A longer fragment of SAF-A encompassing the AAA^+^/RGG domains was purified and the ATPase activity measured in the presence or absence of RNA. Total RNA increased the ATPase activity 2.5 times compared to the absence of RNA (Figure 3B).

To determine whether SAF-A undergoes a conformation change upon ATP-binding, consistent with protein actuation as for other AAA^+^ domain proteins (e.g., DnaA), we measured the change in fluorescence upon nucleotide binding. His-tagged AAA^+^ fragments of SAF-A were treated with increasing concentrations of ATP and the quenching of intrinsic fluorescence of tryptophan and tyrosine residues were measured to reveal a pronounced alteration in structure upon nucleoside binding (Figure 3C). Mutation of the Walker A motif abrogated ATP binding but Walker B mutations had no effect; K_d_ for ATP binding to wild-type SAF-A and Walker B mutant SAF-A were 38 μM and 29 μM, respectively.

ATP binding triggers a change in AAA^+^ domain conformation in other proteins, which then stabilizes subunit-subunit interactions (Erzberger et al., 2006). We hypothesized that SAF-A might form an oligomeric complex upon ATP binding. Further analysis of SAF-A revealed an additional predicted α helix (aa 512–521) after canonical β strand 2. This α helix is analogous to the initiator-specific motif (ISM) observed in DnaA and ORC family proteins (Duderstadt et al., 2011, Erzberger et al., 2006), which guides neighboring AAA domains into a non-planar arrangement, preventing formation of a flat ring (Figure 3D) and required for folding into a spiral oligomer.

### Biochemical Characterization of SAF-A Oligomers

**Figure 4:**
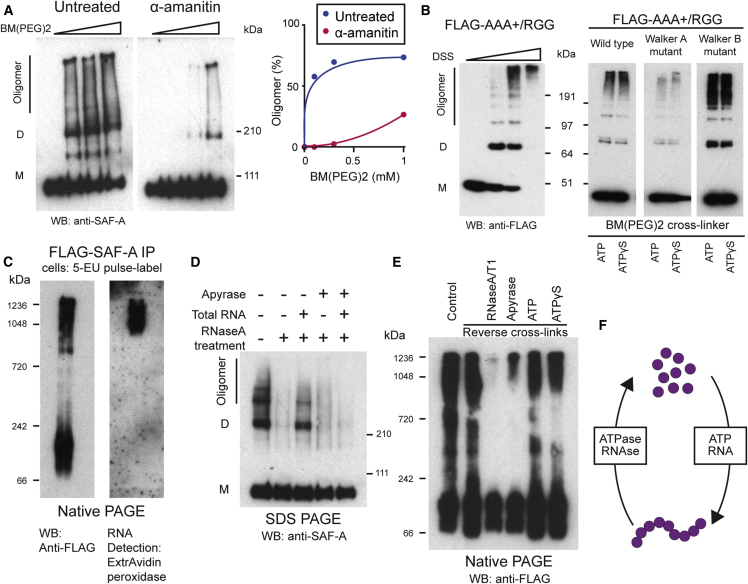
ATP- and RNA-Dependent SAF-A Oligomerization Cycle (A) Left: Western blot for endogenous SAF-A protein extracted from 293T cells treated with or without α-amanitin and stabilized by cross-linking with different concentrations of 1,8-bismaleimido-diethyleneglycol (BM(PEG)2). Proteins were resolved by SDS-PAGE to reveal different SAF-A species (oligomer; D-dimer; M-monomer). Right: Quantification of data in left panel. (B) Left: Western blot for FLAG-tagged SAF-A AAA^+^/RGG protein expressed in 293T cells and stabilized with different concentrations of disuccinimidyl suberate (DSS). Extracted proteins were resolved by SDS-PAGE. Right: SAF-A wild-type, Walker A, or Walker B mutants expressed in 293T cells, treated with ATP or ATPγS and cross-linked with 0.3 mM BM(PEG)2, extracted, and resolved by SDS-PAGE. (C) 293T cells expressing full-length FLAG-tagged SAF-A pulse-labeled with 5-ethynyl uridine (5-EU) and stabilized with BM(PEG)2. SAF-A was extracted, immuno-purified and resolved by native PAGE. Left: Western blot for SAF-A. Right: RNA detection in SAF-A oligomers. Samples were fractionated by native gel electrophoresis, transferred to membrane, and RNA was labeled by conjugating biotin to pre-incorporated 5-EU using click chemistry and detection using avidin-HRP. (D) Western blot for endogenous SAF-A to analyze protein oligomerization. 293T cells pre-treated with RNaseA, then incubated in the presence or absence of total RNA or apyrase and stabilized by cross-linking with BM(PEG)2. Proteins were extracted and resolved by SDS-PAGE (oligomer; D-dimer; M-monomer). (E) Western blot for FLAG-SAF-A to analyze protein de-oligomerization. Cells were cross-linked with dithio-bis-maleimidoethan (DTME) and stabilized SAF-A was immuno-purified. Cross-links were reversed with DTT, then incubated in the presence of RNase, apyrase, or nucleotides and fractionated by native PAGE. (F) Model for the ATP- and RNA-dependent SAF-A (purple) oligomerization cycle. See also Figure S4.

**Figure S4:**
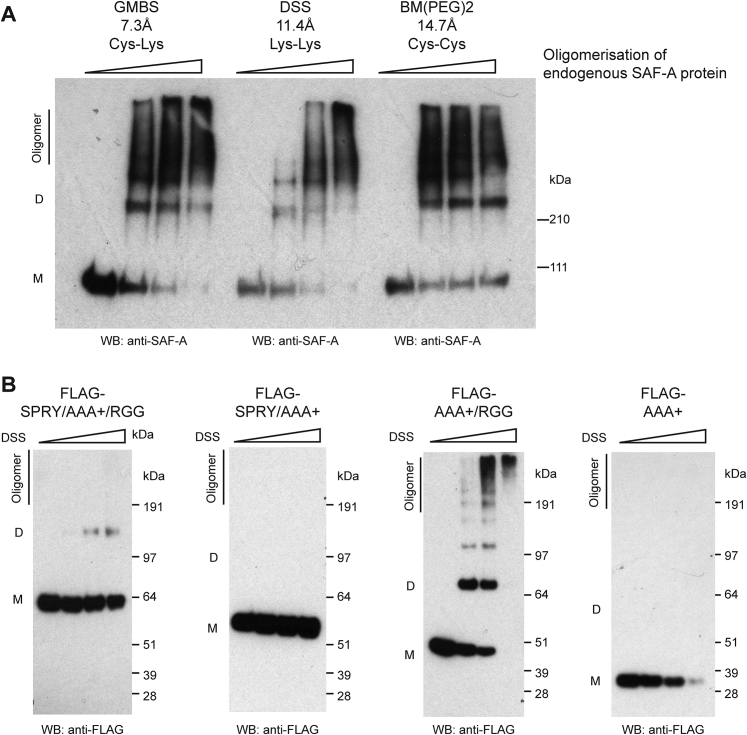
Role of SAF-A Domains in Protein Oligomerization, Related to Figure 4 (A) western blot for endogenous SAF-A protein in 293T cells cross-linked with increasing concentrations of 1,8-bismaleimido-diethyleneglycol (BM(PEG)2), disuccinimidyl suberate (DSS), N-γ-maleimidobutyryl-oxysuccinimide ester (GMBS) and fractionated by SDS-PAGE (Oligomer; D-dimer; M-monomer). (B) western blot for FLAG-tagged SAF-A domains (AAA+; AAA+/RGG; SPRY/AAA+; SPRY/AAA+/RGG) expressed in 293T cells, cross-linked using disuccinimidyl suberate (DSS) and fractionated by SDS-PAGE (Oligomer; D-dimer; M-monomer).

To investigate whether SAF-A forms oligomeric chains, we reasoned that protein-protein cross-linkers would stabilize oligomeric SAF-A structures that could then be isolated from cells. To minimize the likelihood of purifying protein aggregates, we treated cells with cross-linkers with amino acid specificity (BM(PEG)2 [1,8-bismaleimido-diethyleneglycol], Cys-Cys, 14.7 Å; DSS [disuccinimidyl suberate], Lys-Lys, 11.4 Å; GMBS [N-γ-maleimidobutyryl-oxysuccinimide ester], Cys-Lys, 7.3 Å). In contrast to aggregates, oligomeric structures have geometric regularity, so it is less likely that a defined cross-linker will stabilize an aggregate structure. After protein extraction and denaturation, high molecular weight SAF-A species were observed with all cross-linkers consistent with protein oligomerization (Figures 4A and S4A). Transcription inhibition substantially reduced oligomer formation (Figure 4A) suggesting that SAF-A oligomerization is transcription-dependent. To identify critical domains for SAF-A oligomerization, individual FLAG-tagged fragments were expressed in cells, cross-linked, and isolated. The AAA^+^/RGG domain efficiently formed oligomers (Figure 4B) while the SPRY domain was refractory to oligomerization; the RGG RNA binding domain was absolutely required (Figure S4B). In the presence of either ATP or the non-hydrolysable analog ATPγS, the SAF-A AAA^+^/RGG formed high molecular weight oligomeric species indicating that ATP binding maintains the oligomeric form (Figure 4B). In contrast, a AAA^+^/RGG Walker A mutant that is unable to bind ATP showed much less oligomerization, while Walker B mutant fragment-deficient in ATP hydrolysis function stably formed oligomers. These results support the idea that SAF-A undergoes protein oligomerization and hydrolysis similar to DnaA (Duderstadt et al., 2010).

To investigate the molecular basis for cyclical SAF-A oligomerization and hydrolysis, we sought to identify the components involved. Guided by the known DnaA interaction with DNA, we purified oligomeric SAF-A (Figure 4C, left) to identify whether it bound to RNA. We found that newly synthesized RNA specifically bound to oligomeric SAF-A (Figure 4C, right) supporting the idea that SAF-A and RNA form a complex. To analyze the steps required for SAF-A oligomerization, we pre-treated cells with RNaseA to monomerize SAF-A (Figure 4D). Addition of total RNA before cross-linking promoted SAF-A oligomerization, which could be abrogated by apyrase treatment showing that SAF-A oligomerization requires both RNA and ATP. To test SAF-A de-oligomerization, we purified cross-linked oligomeric SAF-A from cells and reversed the cross-links using DTT (Figure 4E). ATP or ATPγS stabilized the oligomers whereas RNase or apyrase treatment promoted monomerization. Together, our data show that SAF-A can undergo cycles of oligomerization promoted by RNA and ATP binding and ATP hydrolysis (Figure 4F).

### SAF-A Forms Oligomers In Vivo

**Figure 5:**
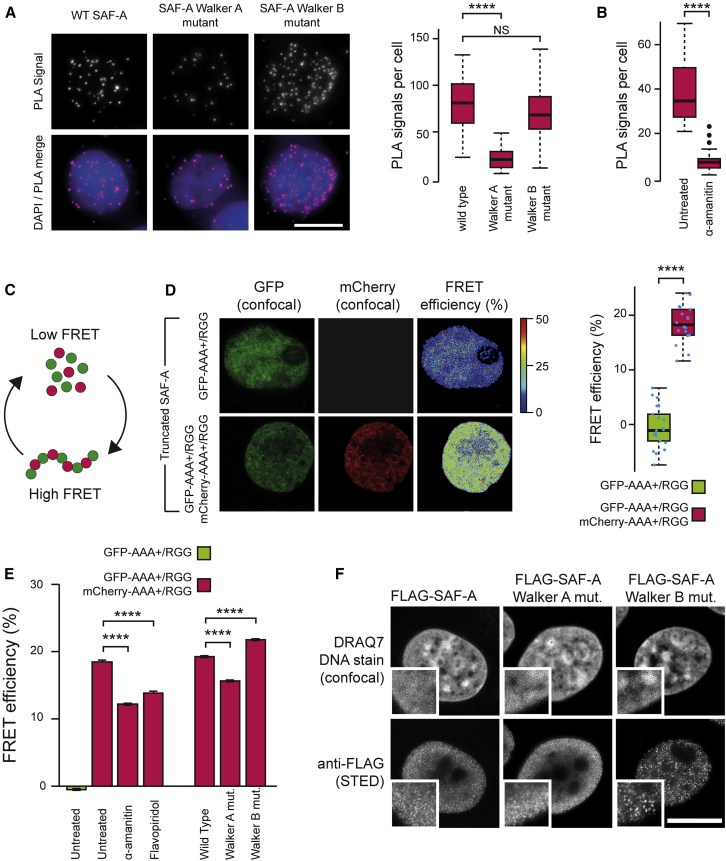
ATP and Transcription-Dependent SAF-A Oligomerization in Cells (A) Left: Proximity ligation assay (PLA) signals in Flp-In T-REx 293 cells depleted of endogenous SAF-A (48 hr) and then doxycycline induced for 24 hr to express FLAG-tagged wild-type SAF-A or Walker A or Walker B mutants and co-expressing T7-tagged wild-type or mutant versions of SAF-A. Scale bar, 10 μm. Right: Quantification of PLA signals. p values are for a Wilcoxon test, n > 30 nuclei for two biological replicates. (B) Boxplot showing number of PLA signals as in (A) for cells treated in the presence or absence of α-amanitin (5 hr treatment). p values for a Wilcoxon test, n = 30 nuclei for two biological replicates. (C) Model depicting the change in Förster resonance energy transfer (FRET) efficiency between monomeric and oligomeric forms of GFP-SAF-A (green) and mCherry-SAF-A (red). (D) Left: Representative confocal images of 293T cells expressing truncated forms of SAF-A: GFP- AAA^+^/RGG or both mCherry-AAA^+^/RGG and GFP-AAA^+^/RGG. Pseudo-colored images show FRET efficiency calculated from fluorescence-lifetime imaging microscopy (FLIM) of individual pixels (see the STAR Methods). Right: Boxplot quantifying average FRET efficiency in GFP-AAA^+^/RGG (green) or GFP-AAA^+^/RGG and mCherry-AAA^+^/RGG (red) cells. p values for Student’s t test, n > 20 nuclei. (E) Bar graph of median pixel-by-pixel FRET efficiency in 293T cells expressing GFP-AAA^+^/RGG (green) or GFP-AAA^+^/RGG and mCherry-AAA^+^/RGG (red). Left: Control cells or cells treated with the transcription inhibitors α-amanitin or flavopiridol. Right: 293T cells depleted of endogenous SAF-A and expressing GFP- and mCherry-tagged wild-type, Walker A, or Walker B SAF-A mutants. p values for Student’s t test. (F). Super-resolution STED microscopy of FLAG-tagged SAF-A protein. Flp-In T-REx 293 cells depleted of endogenous SAF-A (48 hr) and then doxycycline induced to express wild-type or mutant Walker A or Walker B SAF-A and counterstained with DRAQ7. Scale bar, 10 μm. Insets show detail. p values: NS, not significant; ^∗∗∗^p < 0.001; ^∗∗∗∗^p < 0.0001. See also Figure S5.

**Figure S5:**
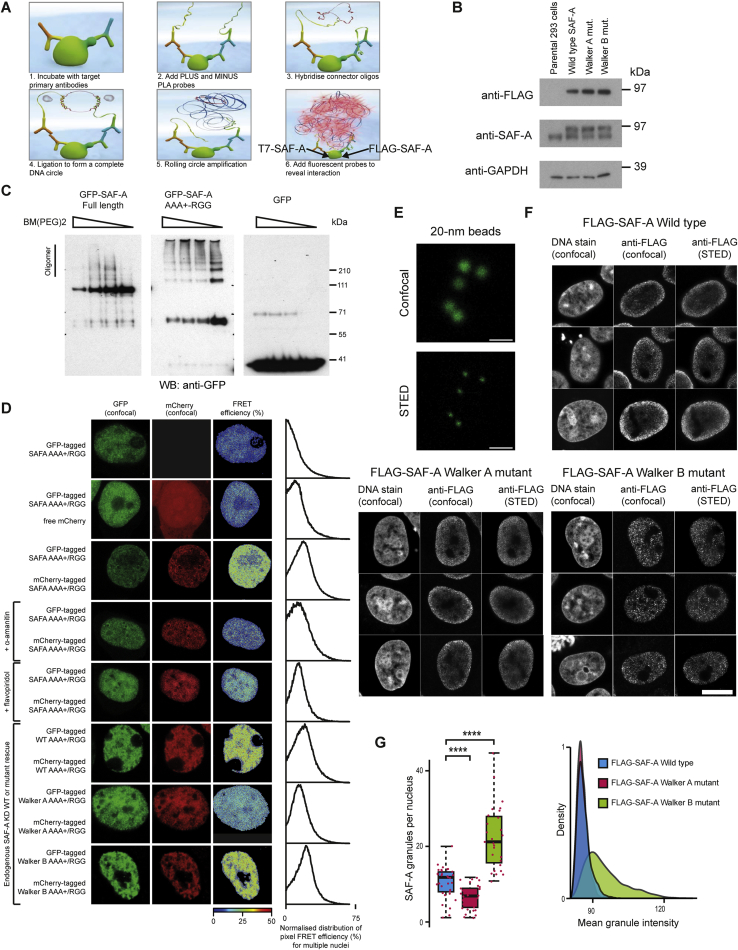
PLA and FLIM-FRET Controls to Analyze SAF-A Interactions, Related to Figure 5 (A) Cartoon representing proximity ligation assay (PLA) to analyze protein-protein associations in cells. (B) western blot for FLAG-tagged wild-type, Walker A or Walker B mutant SAF-A and endogenous SAF-A in Flp-In T-Rex 293 cell lines induced by doxycycline. GAPDH was used as a loading control. (C) western blot for GFP-tagged full length or AAA+/RGG domain SAF-A expressed in 293T cells, cross-linked using 1,8-bismaleimido-diethyleneglycol (BM(PEG)2) and fractionated by SDS-PAGE. (D) Representative confocal images under different experimental conditions in 293T cells expressing fluorescently tagged forms of SAF-A: GFP-AAA+/RGG or GFP-AAA+/RGG with free mCherry or cells expressing both mCherry-AAA+/RGG and GFP-AAA+/RGG and pseudo-colored images showing FRET efficiency calculated from fluorescence-lifetime imaging microscopy (FLIM) of individual pixels. Right, distribution of pixel FRET efficiency for multiple (5 – 20) nuclei. (E) Calibration of STED microscopy using 20-nm fluorescent beads. Scale bar, 500 nm. (F) Super-resolution STED and confocal microscopy of FLAG-tagged wild-type SAF-A or SAF-A Walker A or Walker B mutants in Flp-In T-Rex 293 cells. Cells were depleted of endogenous SAF-A and FLAG tagged proteins induced by doxycycline treatment. Cells were counterstained with DRAQ7. Scale bar, 10 μm. (G) Left, boxplot showing SAF-A granules per nucleus in FLAG-tagged wild-type SAF-A or SAF-A Walker A or Walker B mutants as in Figure 5F and (E). P values for a Wilcoxon test (n > 30 nuclei; ^∗∗∗∗^p < 0.0001). Right, density plot showing mean SAF-A granule intensity in nuclei expressing wild-type or mutant SAF-A (n > 30 nuclei for each sample).

To investigate SAF-A dynamics in live cells, we used three complimentary approaches. First, to assay full-length SAF-A oligomerization in cells, we designed a proximity ligation assay (PLA) to detect SAF-A ↔ SAF-A interactions (Figure S5A). In PLA, proteins are detected with antibodies fused to DNA oligos: close proximity of the proteins allows ligation of the DNA oligos, which can then be amplified and detected by a fluorescent signal. Flp-In T-REx 293 cells were depleted of endogenous SAF-A and then doxycycline treated to express wild-type FLAG-tagged SAF-A or Walker A or Walker B mutants, at a physiological level (Figure S5B), and co-transfected with T7-tagged full-length SAF-A or SAF-A with Walker A or Walker B point mutations. PLA signals indicating SAF-A interactions (Figure 5A) were found in cells expressing wild-type SAF-A or the SAF-A Walker B mutant, while no interactions were observed for the SAF-A Walker A mutant, suggesting that SAF-A oligomerizes in vivo through ATP binding, while ATP hydrolysis is not important for these associations. We next investigated if transcription regulates SAF-A oligomerization in live cells, consistent with its role upstream of SAF-A in model 1 (Figure 2). To directly test this we treated cells with the transcription inhibitor α-amanitin and assayed for SAF-A oligomerization by PLA. After transcription inhibition the number of PLA signals significantly decreased (Figure 5B), concomitant with a reduction in SAF-A oligomeric forms (Figure 4A).

Second, we analyzed SAF-A interactions using FLIM-FRET (fluorescence lifetime imaging microscopy – Förster resonance energy transfer). FLIM-FRET is a well-established approach to detect interactions at the nanometer scale (Llères et al., 2017); we predicted that SAF-A↔SAF-A interactions in oligomers should be detectable by FRET (Figure 5C). Although fluorescent protein tags inhibited the oligomerization of the full-length protein, oligomerization could be observed biochemically for GFP and mCherry-tagged AAA^+^/RGG SAF-A fragments (Figure S5B). We expressed the GFP-tagged AAA^+^/RGG fragment in cells, either on its own (donor only) or in combination with a mCherry-tagged version of the same fragment and measured the fluorescence lifetime of GFP. An interaction between the two differentially labeled fragments is expected to result in energy transfer between the GFP and mCherry tags, associated with a decrease in the fluorescence lifetime of GFP. The difference in GFP lifetime between the donor alone (GFP fragment on its own) and the FRET conditions can then be expressed as FRET efficiency. We found a significant increase in FRET efficiency in cells expressing both GFP and mCherry tagged AAA^+^/RGG fragments, strongly indicative of oligomerization involving the two components (Figure 5D). Consistently with the PLA data, a decrease in FRET efficiency was observed for AAA^+^/RGG Walker A mutant fragments, confirming the importance of ATP binding for oligomerization (Figures 5E and S5C). Fragments carrying Walker B mutations resulted in an increase in FRET efficiency compared to the wild-type, suggesting a defect in the oligomerization cycle and underscoring the role of ATP hydrolysis in SAF-A monomerization. Abolishing transcription, using two different inhibitors, also impeded oligomerization, as evidenced by a decrease in FRET efficiency (Figures 5E and S5C).

Finally, as the FLIM-FRET analysis indicated that the Walker B mutant showed excessive oligomerization, we speculated that ATP hydrolysis might be important for the reversal and dynamics of this process. Consistently, when we examined the staining patterns of FLAG-tagged wild-type SAF-A and the two mutants by super-resolution STED microscopy (Figure S5D), the SAF-A Walker B mutant showed a granular staining pattern indicative of large SAF-A oligomers, which was not observed in the wild-type SAF-A or the SAF-A Walker A mutant (Figures 5F and S5E). Quantification of the granules showed that the Walker B mutant had significantly more and brighter granules than for either wild-type or Walker A mutant (Figure S5F), highly indicative of SAF-A Walker B mutant oligomers continuing to undergo oligomerization, forming large structures. These three pieces of evidence support a model of cyclical SAF-A oligomerization and monomerization in response to transcription and regulated by ATP binding and hydrolysis.

### SAF-A Regulates Chromatin Structure through caRNAs

**Figure 6:**
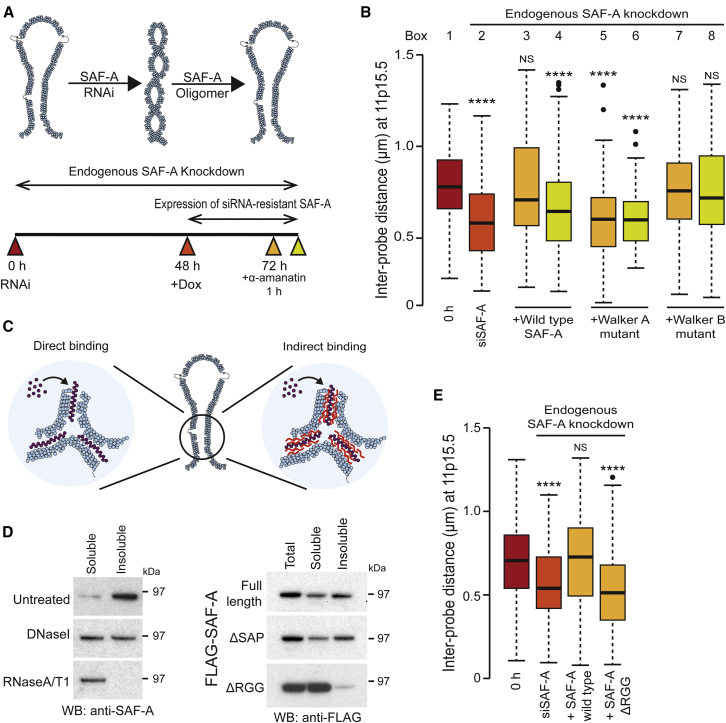
SAF-A Oligomerization Regulates Interphase Chromosome Structure via Chromatin-Associated RNAs (A) Top: Model for chromatin decompaction by SAF-A oligomerization. Bottom: Experimental strategy to test model. Flp-In T-REx 293 cells depleted of endogenous SAF-A (48 hr) and then doxycycline-induced for 24 hr to express wild-type or mutant SAF-A. Samples were taken at times (colored triangles) for analysis. (B) Boxplots showing the distribution of distances between probe pairs at 11p15.5 from a FISH chromatin compaction assay in cells before (0 hr, red rectangle, box 1), after SAF-A depletion (siSAF-A, dark orange rectangle, box 2), and after re-expression of siRNA-resistant wild-type SAF-A, Walker A, or Walker B mutants in the absence (pale orange rectangles, boxes 3, 5, 7) or presence (green rectangles, boxes 4, 6, 8) of α-amanitin (n = 100 for two biological replicates). Colored bars correspond to time points shown in (A). (C) Model depicting direct or indirect binding of SAF-A (purple) to chromatin (blue) in the presence of caRNAs (red). (D) Left: Western blot showing extraction of SAF-A into soluble and insoluble fractions from cells before and after treatment with RNase A/T1 or DNaseI. Right: Western blot showing FLAG-tagged full length SAF-A or SAF-A truncated at the N (ΔSAP) or C (ΔRGG) termini extracted into soluble and insoluble fractions. (E) Boxplots as described in (B) showing effect of re-expressing SAF-A lacking the RGG-domain on large scale chromatin compaction. Colored bars correspond to time points in (A). p values for a Wilcoxon test: NS, not significant; ^∗∗∗∗^p < 0.0001. See also Figure S6 and Table S1.

**Figure S6:**
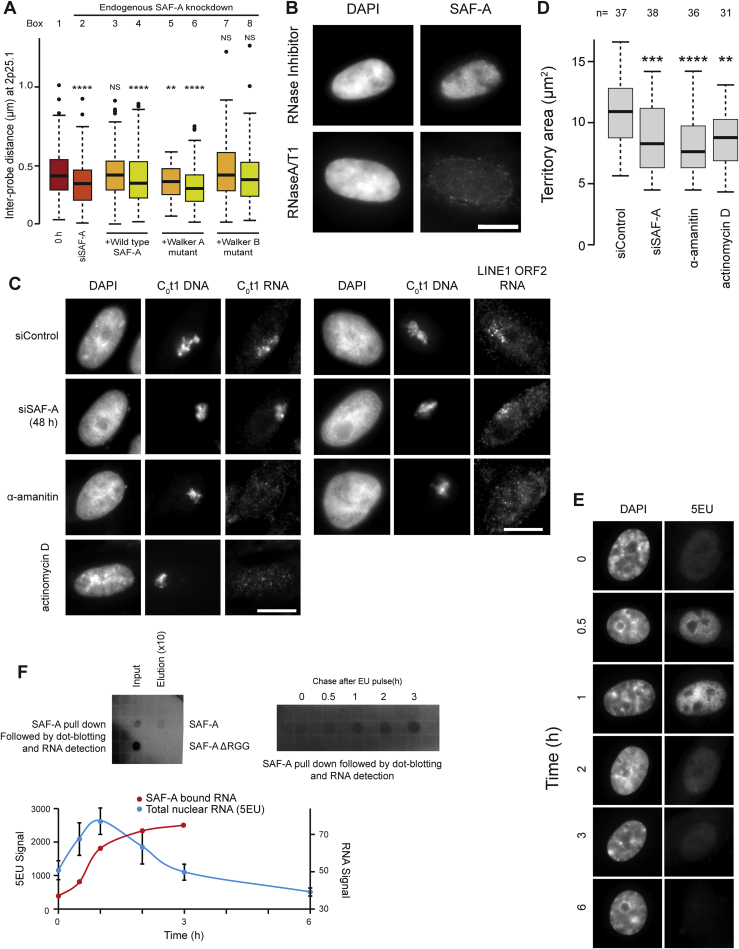
SAF-A Oligomerization Regulates Interphase Chromatin Structure via Chromatin-Associated RNAs but Is Not Required to Maintain C_0_t1 and LINE-1 RNA, Related to Figure 6 (A) Boxplots showing the distribution of distances between probe pairs at 2p25.1 from a FISH chromatin compaction assay in cells before (0 hr, red rectangle, box 1), after SAF-A depletion (siSAF-A, dark orange rectangle, box 2) and after re-expression of siRNA-resistant wild-type SAF-A, Walker A or Walker B mutants in the absence (pale orange rectangles, box 3, 5, 7) or presence (green rectangles, box 4, 6, 8) of α-amanitin (n > 100). Colored bars correspond to time points shown in Figure 6A. (B) Immunofluorescence for SAF-A in Triton X-100 extracted RPE1 cells treated with RNaseA/T1, counterstained with DAPI. Scale bar, 10μm. (C) DNA-FISH for human chromosome 3 (HSA3, C_0_t1 DNA probe) in a human-hamster hybrid cell line (GM10253A) that stably carries HSA3 in conjunction with RNA-FISH to assay for the binding and distribution of caRNAs (C_0_t1 RNA probe) or LINE-1 ORF2 RNA in control cells (siControl) and cells depleted for SAF-A (siSAF-A) and cells treated with the transcription inhibitors α-amanitin or actinomycin D. Scale bars, 10 μm. (D) Boxplot showing area occupied by HSA3 territory (C_0_t1 DNA probe) as in panel C in GM10253A control cells (siControl), cells depleted for SAF-A (siSAF-A) and cells treated with the transcription inhibitors α-amanitin or actinomycin D (5 h). P values for a Wilcoxon test (n > 30 nuclei; ^∗∗∗∗^p < 0.0001). (E) Fluorescence microscopy in RPE1 cells pre-treated with low dose (50 ng/ml) actinomycin D (60 min) and pulse labeled (15 min) with 5-ethynyl uridine (5-EU), fixed at the indicated time post 5-EU treatment. 5-EU was conjugated to Cy5 in a click chemistry reaction for visualization. (F) Top left, dot blotting of RNA co-purified with FLAG-tagged full length SAF-A or SAF-A truncated at the C terminus to remove the RGG domain; Flp-In T-REx 293 cells were doxycycline induced for 24 hr to express wild-type or mutant SAF-A. RNA was labeled (15 min) with 5-EU followed by crosslinking with BM(PEG)2 and immune-purified with FLAG antibody, and conjugated using click chemistry with a biotin azide and detected by streptavidin-conjugated horseradish peroxidase and chemiluminescence. Top right, dot blot showing RNA co-purified with FLAG-tagged full length SAF-A. Bottom, quantification of 5-EU and RNA signals shown in panel E and F.

We next tested whether SAF-A oligomerization was required for regulation of large-scale chromatin structures by examining 11p15.5 (Figure 6B) or 2p25.1 (Figure S6A) compaction following rescue experiments with RNAi-resistant wild-type SAF-A, or SAF-A encoding Walker A or Walker B mutants at different time points (Figure 6A, colored triangles). As predicted, wild-type SAF-A was able to rescue the compacted phenotype and efficiently decompact chromatin (Figure 6B, box 3). Expression of the Walker A mutant was unable to rescue the chromatin state (Figure 6B, box 5) showing that SAF-A oligomerization is required for chromatin decompaction. Conversely, the Walker B mutant was able to decompact the gene-rich region (Figure 6B, box 7), indicating that oligomerization, and not the dynamic cycling between oligomeric and monomeric states, is essential for decompacting large-scale chromatin structure. In the opposite experiment, we tested whether SAF-A monomerization was necessary for chromatin compaction, taking advantage of the ability of α-amanitin to compact chromatin (Figure 1F): expression of the SAF-A protein in the presence of α-amanitin (Figure 6B, box 8) was able to drive chromatin compaction (Figure 6B, box 4), however, the Walker B mutant was unable to drive chromatin compaction (Figure 6B, box 8) consistent with ATP-hydrolysis being required to cycle from an oligomeric to monomeric state to compact chromatin. Thus, we conclude that SAF-A oligomer assembly and disassembly regulates large-scale chromatin structure.

We next addressed how SAF-A interacted with chromatin to regulate large-scale chromatin structures. We hypothesized two potential modes of interaction: SAF-A could either be directly bound to chromatin, or it might bind indirectly, interacting with RNAs (Figure S2B) through the RGG domain (Figure 2A). In pilot experiments, we found that unlike positive controls, DNA was not co-precipitated in chromatin immunoprecipitation (ChIP) assays with antibodies against SAF-A, suggesting the protein was not directly bound to chromatin. In contrast, cross-linking immunoprecipitation sequencing (CLIP-seq) experiments have shown that SAF-A is able to bind to virtually all classes of regulatory RNA (Xiao et al., 2012).To analyze the mode of SAF-A binding, nuclear samples were treated with RNaseA/T1 or DNaseI before extraction into a soluble and insoluble (chromatin-associated) state; RNase treatment significantly abolished SAF-A chromatin binding, while DNaseI only had a limited effect (Figure 6D). To further explore the mechanics of SAF-A/RNA interaction with chromatin, we used an approach developed by Hall et al. (2014) based on the observation that caRNAs, detected by RNA-FISH with a human C_0_t1 probe in human-hamster hybrids, remain associated with the chromosome territory from where they are transcribed, in an unstable manner (t_1/2_ = 1 hr, Figure S6E). SAF-A depletion did not affect C_0_t1 RNA binding to chromosomes (Figure S6C) while transcription inhibition triggered a rapid loss of C_0_t1 or LINE-1 signal and chromosome territory compaction (Figure S6D), consistent with SAF-A binding to chromatin via caRNAs. We also found that SAF-A bound to newly transcribed caRNA through its RGG domain (Figure S6F) and further tested whether this binding was required for chromatin decompaction using the approach developed in Figure 6A. Re-expression of SAF-A ΔRGG was unable to decompact chromatin structures (Figure 6E) indicating that SAF-A oligomerization regulates large-scale chromatin structures through an interaction with caRNAs.

### SAF-A Is Required for Chromosome Stability

**Figure 7:**
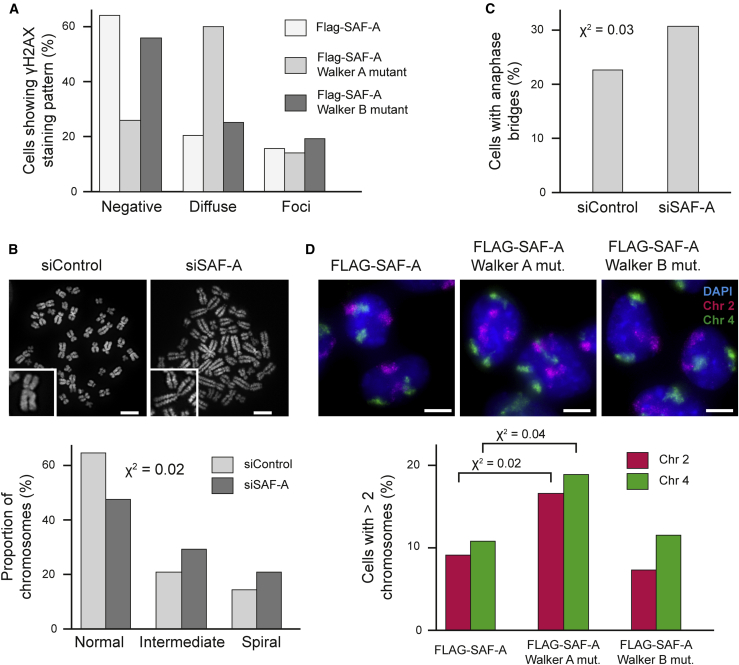
Disruption of SAF-A-Dependent Large-Scale Chromatin Structure Causes Genome Instability (A) Quantification of the frequency of diffuse and focal γ-H2AX signals. Flp-In T-REx 293 cells were depleted of endogenous SAF-A (48 hr) and then doxycycline induced for 24 hr to express wild-type or mutant SAF-A. (B) Top: Representative images of mitotic chromosomes prepared from RPE1 cells (siControl) or cells depleted of SAF-A (siSAF-A) and stained with DAPI. Scale bars, 5 μm. Bottom: Quantification of chromosome morphology. (C) Frequency of anaphase bridges observed after staining with BLM in RPE1 cells (siControl, n = 53) or cells depleted of SAF-A (siSAF-A, n = 39). (D) Top: DNA FISH for human chromosome 2 (HSA2) and 4 (HSA4) territories in Flp-In T-REx 293 cells depleted of endogenous SAF-A, with wild-type or Walker A or B SAF-A mutants re-expressed. Nuclei were counterstained with DAPI (blue). Scale bars, 10 μm. Bottom: Quantification of chromosome territory number for HSA2 and HSA4 (n > 150 nuclei; p values for a chi-square test). See also Figure S7.

**Figure S7:**
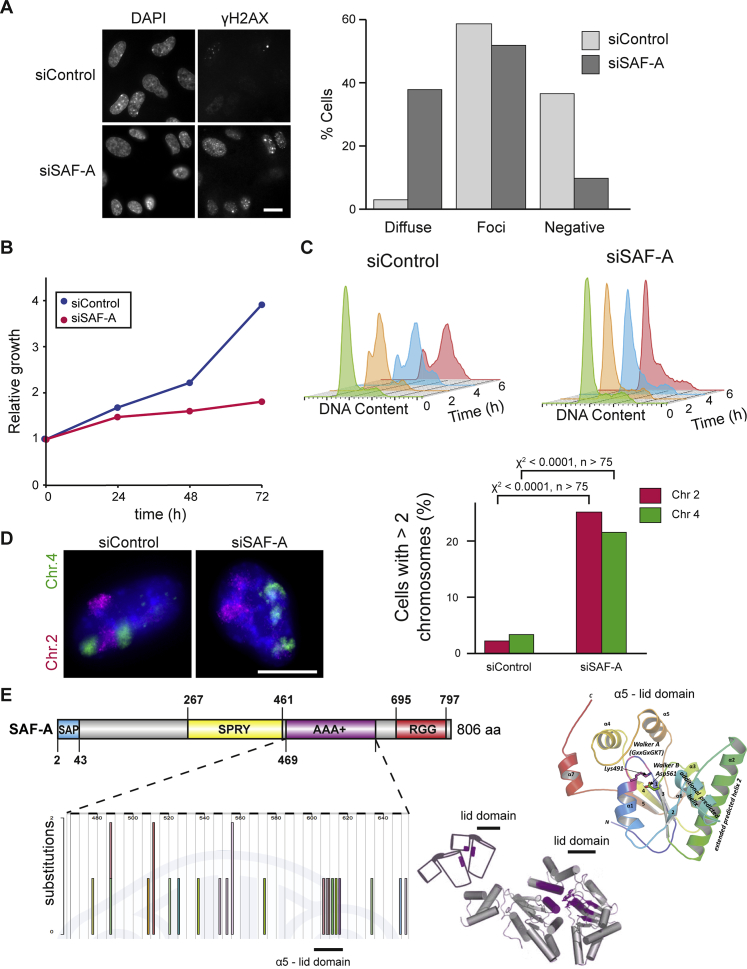
Characterization of Genome Instability and Distribution of SAF-A Cancer Mutations, Related to Figure 7 (A) Left, immunofluorescence staining for γ-H2AX in RPE1 control cells (siControl) or cells depleted of SAF-A (siSAF-A). Scale bar, 10 μm. Right, quantification of the frequency of diffuse and focal γ-H2AX signals, data representative of two biological replicates. (B) Growth curve for RPE1 cells (siControl) and cells depleted of SAF-A (siSAF-A) across a 72 hr time period. (C) Cell cycle profile for control cells (siControl) and cells knocked down for SAF-A (siSAF-A). After knock-down cells were synchronized using aphidicolin for 24 hr and released. Aliquots were taken at 0, 2, 4, 6 hr, stained with propidium iodide and the DNA content analyzed by flow cytometry. (D) Left, FISH for human HSA2 and HSA4 chromosome territories in RPE1 control cells (siControl) or cells depleted of SAF-A (siSAF-A). Scale bar, 10 μm. Right, quantification of chromosome territory number for HSA2 and HSA4 (n > 75, p values are for a Chi squared test). (E) Left, distribution of SAF-A AAA+ mutations from COSMIC (Catalogue Of Somatic Mutations In Cancer) in the SAF-A AAA+ domain. Right, diagram showing position of the AAA+ lid domain.

Unexpectedly, our data indicates that altering large-scale chromatin structure, by depletion of SAF-A, only has limited impact on transcription (Figures 2 and S2). However, we speculated that interphase chromatin structure disruption would have an effect on genome integrity. We observed diffuse phosphorylated H2AX (γ-H2AX) signals in SAF-A-depleted RPE1 cells (Figure S7A), a response reflective of nuclear stress (Meyer et al., 2013). Diffuse γ-H2AX signals appeared 48 hr after an alteration in large-scale chromatin structure suggesting that irregular chromatin triggers the response. We also characterized the effect of Walker A or Walker B mutations on genomic stability: only the oligomerization-deficient Walker A mutant promoted the accumulation of diffuse γ-H2AX pattern (Figure 7A) suggesting that inhibition of oligomerization and enforced chromatin compaction triggers a response, indicative of cellular mechanisms that monitor chromatin structure integrity. Spiral-shaped and distorted metaphase chromosomes were also observed in SAF-A-depleted cells, which also showed reduction in viability, G1/S phase arrest (Figures 7B and 7C), chromosomal segregation defects, and the formation of anaphase bridges staining positive for the BLM protein (Figure 7C). To assess the long-term impact of an alteration in chromatin structure on chromosomal instability, we performed chromosome painting FISH for HSA2 and HSA4 (Figure S7D). Control cells predominantly had two copies of HSA2 and HSA4 while 25% and 21%, respectively, have more than three chromosome signals after the depletion of SAF-A, indicating that an alteration in interphase chromatin structure causes chromosomal instability. A similar effect could be observed in cells expressing the SAF-A Walker A mutant, but not the Walker B mutant, indicating that large-scale chromatin structure, regulated by SAF-A oligomerization, is essential for chromosome stability (Figure 7D).

## Discussion

We have demonstrated a previously unknown role for the abundant nuclear protein SAF-A in regulating interphase chromosome structure via oligomerization with caRNAs. ATP binding through the SAF-A AAA^+^ domain mediates oligomerization, while the RNA-binding domain of SAF-A directly links protein assembly with caRNAs. Our preliminary data indicates that caRNAs have a short half-life (∼1 hr) in the nucleus, and the caRNA pool is rapidly turned over suggesting that caRNAs should be considered as a dynamic nuclear compartment that is involved in chromatin structure regulation. It is therefore possible that caRNAs, generated by normal transcription, decompact large-scale chromatin structures facilitating their transport from the nucleus.

Expanding both the classical concept of a nuclear matrix underpinning interphase chromatin domains and more recent observations of RNA as a fundamental chromatin component (Hall and Lawrence, 2016), we suggest that SAF-A, together with heterogeneous caRNAs forms a dynamic chromatin mesh that regulates chromatin structure. Unlike previously envisioned static models of the nuclear matrix, this process is dynamic and responsive to transcription. From our studies, it is not clear what structure SAF-A adopts. However, the protein shows compelling similarity to DnaA protein, and the presence of an ISM suggests it might be a spiral oligomer (Erzberger et al., 2006). The underlying regulatory mechanisms for triggering SAF-A oligomerization are unclear, but SAF-A has a number of predicted phosphorylation sites and interacts with known kinases (Britton et al., 2014, Douglas et al., 2015). In addition, a SAF-A homolog, HNRNPUL1, shows 60% similarity, but there is no evidence for the presence of a Walker A or Walker B motif raising the possibility that it might function as a regulatory protein to “cap” growing SAF-A chains.

An insight into the functional interplay between SAF-A and chromatin could be provided by recent simulation experiments suggesting that non-specific binding-induced-attraction is sufficient to generate local chromatin domains or chromatin micro-bodies (Brackley et al., 2016). Formation is driven by protein-chromatin interactions mediated by RNA that could partition the genome into functionally open or closed domains, observed by chromosome conformation capture techniques, providing an evolutionary constraint for maintaining gene-rich domains together (Ghanbarian and Hurst, 2015). Surprisingly, this model does not require explicit boundary elements suggesting that evolutionary domains could divide or merge depending on local transcription. It is also tempting to speculate that transcription ripples that occur over 100 kb in mammalian cells could limit the maximum size of functional domains (Ebisuya et al., 2008). The regulation of chromatin micro-bodies is unknown but could be regulated by the cyclical ATP-dependent polymerization of SAF-A, while extensive RNA-chromatin interactions will provide a “glue” to stabilize local chromatin structures.

Previous reports show that SAF-A is essential for mouse embryonic development (Roshon and Ruley, 2005, Ye et al., 2015), and numerous mutations have been reported in the AAA^+^ domain of SAF-A in the cancer genome sequencing projects with a pronounced cluster in the AAA^+^ lid domain (Figure S7E). It has also recently been reported that haploinsufficiency for SAF-A causes impaired neurological development in humans (Deciphering Developmental Disorders Study, 2015). Consistent with these pathological effects, our data shows that maintenance of interphase chromosome structure by SAF-A is necessary to maintain chromosome stability and aberrant chromatin structure triggers a DNA damage response (Figure 7). This suggests the mammalian genome has mechanisms for sensing changes in large-scale chromatin structure. This is signaled by an ATM/ATR-dependent response that leads to H2AX phosphorylation and a rapid cell-cycle arrest. While we show that large-scale chromatin structures are secondary to transcription, our data highlights that maintenance of chromosome organization is necessary for chromosome stability, implicating chromatin architecture as a guardian of the genome.

## STAR★Methods

### Key Resources Table

**Table:** 

REAGENT or RESOURCE	SOURCE	IDENTIFIER
**Antibodies**		

anti-FLAG M2 (1:5000 for western blotting, 1:500 for immunofluorescence)	Sigma-Aldrich	Cat# F1804; RRID: AB_262044
anti-FLAG M2 Affinity Gel	Sigma-Aldrich	Cat# A2220; RRID: AB_10063035
anti-FLAG (1:500 for PLA)	Sigma-Aldrich	Cat# F7425; RRID: AB_439687
anti-T7 (1:500 for PLA)	Millipore	Cat# 69522
anti-GFP (1:5000 for western blotting)	Abcam	Cat# ab290; RRID: AB_303395
anti-Histone H3 (1:50000 for western blotting)	Abcam	Cat# ab1791; RRID: AB_302613
anti-γ-H2AX (1:1000 for immunofluorescence)	Millipore	Cat# 05-636
anti-hnRNP A1 (1:3000 for western blotting)	Invitrogen	Cat# PA5-19431
anti-hnRNP C (1:3000 for western blotting)	Santa Cruz	Cat# sc32308; RRID: AB_627731
anti-GAPDH (1:5000 for western blotting)	Cell Signaling	Cat# 2118L; RRID: AB_561053
anti-SAF-A 3G6 (1:5000 for western blotting)	Abcam	Cat# ab10297
anti-SAF-A for IF (1:200 for immunofluorescence)	Bethyl	Cat# A300-690A
anti-Ki67 (1:300 for immunofluorescence)	Abcam	Cat# ab15580; RRID: AB_443209
anti-sc35 (1:300 for immunofluorescence)	Abcam	Cat# ab11826; RRID: AB_298608
anti-BLM (1:500 for immunofluorescence)	Santa Cruz	Cat# sc7790; RRID: AB_2243489
Goat anti-Mouse IgG (H+L) Highly Cross-Adsorbed Secondary Antibody, Alexa Fluor 488	ThermoFisher	Cat# A11029; RRID: AB_138404
Donkey anti-Rabbit Texas Red	Jackson ImmunoResearch	Cat# 711-075-152
Goat anti-Mouse IgG (H+L) Cross-Adsorbed Secondary Antibody, Alexa Fluor 647	ThermoFisher	Cat# A21235; RRID: AB_141693

**Chemicals, Peptides, and Recombinant Proteins**		

ExtrAvidin−Peroxidase	Sigma-Aldrich	Cat# E2886
3X FLAG Peptide	Sigma-Aldrich	Cat# F4799
α-amanitin	Sigma-Aldrich	Cat# A2263
Flavopiridol	Sigma-Aldrich	Cat# F3055
Actinomycin D	Sigma-Aldrich	Cat# A4262
DRB	Sigma-Aldrich	Cat# D1916
Hygromycin B	Roche	Cat# 10843555001
Puromycin	ThermoFisher	Cat# A1113803
Doxycycline	Sigma-Aldrich	Cat# D9891
Lipofectamine RNAiMAX Reagent	ThermoFisher	Cat# 13778150
Lipofectamine 2000 Reagent	ThermoFisher	Cat# 11668019
Lipofectamine 3000 Reagent	ThermoFisher	Cat# L3000015
TURBO DNase	ThermoFisher	Cat# AM2239
DNaseI	NEB	Cat# M0303S
Nuclease P1	Sigma-Aldrich	Cat# N8630
RNase A/T1	Ambion	Cat# AM2286
PureLink RNase A	ThermoFisher	Cat# 12091039
RNasin Plus RNase Inhibitor	Promega	Cat# N2611
Apyrase	NEB	Cat# M0398S
Disuccinimidyl suberate (DSS)	ThermoFisher	Cat# 21655
1,8-bismaleimido-diethyleneglycol (BM(PEG)2)	ThermoFisher	Cat# 22336
N-γ-maleimidobutyryl-oxysuccinimide ester (GMBS)	ThermoFisher	Cat# 22309
Dithio-bis-maleimidoethane (DTME)	ThermoFisher	Cat# 22335
L-cysteine	Sigma-Aldrich	Cat# 168149
NuPAGE LDS sample buffer	ThermoFisher	Cat# NP0007
7% tris-acetate gels	ThermoFisher	Cat# EA03555BOX
NuPAGE Tris-Acetate SDS Running Buffer (20X)	ThermoFisher	Cat# LA0041
NuPAGE 4-12% Bis-Tris Protein Gels	ThermoFisher	Cat# NP0323BOX
Bolt 8% Bis-Tris Plus Gels	ThermoFisher	Cat# NW00085BOX
NuPAGE MOPS SDS Running Buffer (20X)	ThermoFisher	Cat# NP0001
NativePAGE 4-16% Bis-Tris Protein Gels	ThermoFisher	Cat# BN1004BOX
NativePAGE Running Buffer Kit	ThermoFisher	Cat# BN2007
NativePAGE Sample Prep Kit	ThermoFisher	Cat# BN2008
Immobilon-P Membrane, PVDF, 0.45 μm	Merck Millipore	Cat# IPVH00010
SuperSignal West Femto Maximum Sensitivity Substrate	ThermoFisher	Cat# 34094
SuperSignal West Pico Chemiluminescent Substrate	ThermoFisher	Cat# 34080
5-ethynyl uridine (5-EU)	BaseClick	Cat# BCN-003-5
4-thiouridine	Sigma-Aldrich	Cat# T4509
Thymidine	Sigma-Aldrich	Cat# T9250
Adenosine 5′-triphosphate disodium salt hydrate	Sigma-Aldrich	Cat# A2383
Guanosine 5′-triphosphate sodium salt hydrate	Sigma-Aldrich	Cat# G8877
Adenosine 5′-[γ-thio]triphosphate tetralithium salt	Sigma-Aldrich	Cat# A1388
Biotin Azide (PEG4 carboxamide-6-Azidohexanyl Biotin)	ThermoFisher	Cat# B10184
Alexa Fluor 647 Azide, Triethylammonium Salt	ThermoFisher	Cat# A10277
Digoxigenin-11-UTP	Roche	Cat# 00000001109−3088910
Biotin-16-dUTP	Roche	Cat# 00000001109−3070910
DRAQ7	Abcam	Cat# ab109202

**Critical Commercial Assays**		

Duolink In Situ PLA Probe Anti-Mouse PLUS Affinity purified Donkey anti-Mouse IgG (H+L)	ThermoFisher	Cat# DUO92001
Duolink In Situ PLA Probe Anti-Rabbit MINUS Affinity purified Donkey anti-Rabbit IgG (H+L)	ThermoFisher	Cat# DUO92005
Duolink In Situ Detection Reagents Red	ThermoFisher	Cat# DUO92008
Click-iT EdU Alexa Fluor 647 Imaging Kit (for 5EU labeling)	ThermoFisher	Cat# C10337
Click-iT Nascent RNA Capture Kit	ThermoFisher	Cat# C10340
BIOMOL Green	Enzo Life Science	Cat# BML-AK111-0250
Ribo-Zero rRNA Removal Kit (Human/Mouse/Rat)	illumina	Cat# MRZH116
RNeasy Mini kit	QIAGEN	Cat# 74104
NEBNext Ultr RNA Library Prep Kit for Illumina	NEB	Cat# E7530S
High Sensitivity DNA Kits	Agilent Genomics	Cat# 5067-4626

**Deposited Data**		

RNA-seq data	This paper	GEO: GSE98541

**Experimental Models: Cell Lines**		

Human: hTERT-RPE1 cell	ATCC	Cat# ATCC-CRL-4000
Human: 293T	ATCC	Cat# ATCC-CRL-3216
Human: FlpIn T-REx 293 Cell line	ThermoFisher	Cat# R78007
Human-Hamster hybrid: GM10253	Coriell Institute	N/A
Human: FlpIn T-REx 293 FLAG-SAF-A	This paper	N/A
Human: FlpIn T-REx 293 FLAG-SAF-A Walker A mut.	This paper	N/A
Human: FlpIn T-REx 293 FLAG-SAF-A Walker B mut.	This paper	N/A
Human: FlpIn T-REx 293 FLAG-SAF-A ΔSAP	This paper	N/A
Human: FlpIn T-REx 293 FLAG-SAF-A ΔRGG	This paper	N/A

**Oligonucleotides**		

Stealth RNA target sequence: SAF-A CCUGGGAAUCGTGGCGGATATAATA	ThermoFisher	Cat# HSS104917
Stealth RNA target sequence: hnRNP C GCUUUGCCUUCGUUCAGUAUGUUAA	ThermoFisher	Cat# HSS179304
Stealth RNA siRNA Negative Control, Med GC	ThermoFisher	Cat# 12935300
Custom MyTags libraries	MYcroarray	N/A

**Recombinant DNA**		

cDNA for SAF-A	Genome Cube	Cat# IRATp970D1041D
Plasmid: FlpIn 3xFLAG-SAF-A	This paper	N/A
Plasmid: FlpIn 3xFLAG-SAF-A Walker A mut.	This paper	N/A
Plasmid: FlpIn 3xFLAG-SAF-A Walker B mut.	This paper	N/A
Plasmid: FlpIn 3xFLAG-SPRY/AAA+/RGG	This paper	N/A
Plasmid: FlpIn 3xFLAG-SPRY/AAA+	This paper	N/A
Plasmid: FlpIn 3xFLAG-AAA+/RGG	This paper	N/A
Plasmid: FlpIn 3xFLAG-AAA+/RGG Walker A mut.	This paper	N/A
Plasmid: FlpIn 3xFLAG-AAA+/RGG Walker B mut.	This paper	N/A
Plasmid: FlpIn 3xFLAG-AAA+	This paper	N/A
Plasmid: FlpIn 3xFLAG-SAF-A ΔSAP	This paper	N/A
Plasmid: FlpIn 3xFLAG-SAF-A ΔRGG	This paper	N/A
Plasmid: FlpIn GFP-SAF-A	This paper	N/A
Plasmid: FlpIn GFP-AAA+/RGG	This paper	N/A
Plasmid: FlpIn GFP-AAA+/RGG Walker A mut.	This paper	N/A
Plasmid: FlpIn GFP-AAA+/RGG Walker B mut.	This paper	N/A
Plasmid: FlpIn mCherry-AAA+/RGG	This paper	N/A
Plasmid: FlpIn mCherry-AAA+/RGG Walker A mut.	This paper	N/A
Plasmid: FlpIn mCherry-AAA+/RGG Walker B mut.	This paper	N/A
Plasmid: T7-SAF-A	This paper	N/A
Plasmid: T7-SAF-A Walker A mut.	This paper	N/A
Plasmid: T7-SAF-A Walker B mut.	This paper	N/A
Plasmid: pET32a-AAA+	This paper	N/A
Plasmid: pET32a-AAA+ Walker A mut.	This paper	N/A
Plasmid: pET32a-AAA+ Walker B mut.	This paper	N/A
Plasmid: pET32a-AAA+RGG	This paper	N/A
Plasmid: FlpIn LINE1 ORF2	This paper	N/A
Plasmid: mCherry-vector	This paper	N/A
Human Cot-1 DNA	ThermoFisher	Cat# 15279011
pOG44	ThermoFisher	Cat# V600520
pSuper-puro vector harboring hnRNP A1 target sequence	Guil et al., 2006	N/A
Fosmids	See Table S1	BacPac resources
Human Chromosome 2 painting probe	Cytocell	Cat# LPP-02R-A
Human Chromosome 4 painting probe	Cytocell	Cat# LPP-04G-A
Human Chromosome 18 painting probe	MetaSystems	Cat# D-0318-050-OR
Human Chromosome 19 painting probe	MetaSystems	Cat# D-0319-050-FI

**Software and Algorithms**		

Woolz image processing system	Armit et al., 2015	https://github.com/ma-tech/Woolz
MAPaint	Armit et al., 2015	N/A
SymPhoTime v5.4.4	PicoQuant	https://www.picoquant.com/products/category/software
TopHat	Trapnell et al., 2012	https://ccb.jhu.edu/software/tophat/index.shtml
Cufflinks	Trapnell et al., 2012	https://ccb.jhu.edu/software/tophat/index.shtml
Bowtie2	Langmead and Salzberg, 2012	http://bowtie-bio.sourceforge.net/index.shtml
iVision	BioVisionTechnologies	http://www.biovis.com/ivision.html
Leica Application Suite Advanced Fluorescence (LAS AF) Software	Leica Microsystems	http://www.leica-microsystems.com/home/
SMART	Schultz et al., 1998	N/A
MetaPrDOS	Ishida and Kinoshita, 2008	http://prdos.hgc.jp/cgi-bin/meta/top.cgi
PHYRE-2	Kelley et al., 2015	http://www.sbg.bio.ic.ac.uk/∼phyre2/html/page.cgi?id=index
Modeler 9v12	Eswar et al., 2007	https://salilab.org/modeller/manual/
PyMol	Schrödinger	http://www.pymol.org
Pro-origami	Stivala et al., 2011	http://munk.csse.unimelb.edu.au/pro-origami/
EsPript v3	Gouet et al., 1999	http://espript.ibcp.fr/ESPript/ESPript/

**Other**		

Prescision Coverslips	ZEISS	Cat# 474030-9000-000
FluoSpheres Carboxylate-Modified Microspheres, 0.02 μm, yellow-green fluorescent (505/515), 2% solids	ThermoFisher	Cat# F8787
HisTrap HP, 1 × 5 ml	GE Healthcare Life Sciences	Cat# 17-5248-01
CM Sepharose	Sigma-Aldrich	Cat# CCF100 Sigma

### Contact for Reagent and Resource Sharing

Further information and requests for reagents should be directed to and will be fulfilled by the Lead Contact, Nick Gilbert (nick.gilbert@ed.ac.uk).

### Experimental Model and Subject Details

#### Cell culture

RPE1, 293T and FlpIn-T-REx-293 cells (ThermoFisher) were cultured as described previously (Naughton et al., 2013). GM10253A hybrid cells (Coriell) were cultured in RPMI1640 supplemented with 3 mM L-glutamine, 10% fetal calf serum, penicillin (100 U.ml^-1^), streptomycin (100 μg.ml^-1^) and phenol red (8.1 mg.l^-1^). Transcription was blocked by adding α-amanitin (50 μg.ml^−1^), flavopiridol (100 μM), actinomycin D (500 nM) or DRB (50 μM) to cells for the times indicated. Flp-In T-REx 293 was used to establish stable cell lines for inducible expression of FLAG-tagged SAF-A and their derivatives by transfecting a modified pcDNA5/FRT Expression vector (ThermoFisher) harboring N-terminal 3 × FLAG tagged SAF-A or its derivatives, and pOG44 (ThermoFisher) encoding Flp recombinase. After selection with 50 μg.ml^-1^ hygromycin B (ThermoFisher) for 2 weeks, the cells were treated with 1 μg.ml^-1^ doxycycline for 24 hr for inducing expression of proteins.

### Method Details

#### Plasmids

cDNA for human SAF-A was obtained from Genome Cube (IRATp970D1041D). Truncation and point mutations of SAF-A were constructed by a standard PCR cloning strategy and inserted into the corresponding vectors with indicated tags. All plasmids were verified by DNA sequencing. siRNA resistant SAF-A was constructed by introducing silent mutations into the nucleotide sequence (2113-2124 bp) 5′-CCTGGGAATCGT-3′ as follows: 5′- CCaGGaAAcCGa −3′. The SAF-A cDNA and derivatives were inserted into a modified pcDNA5/FRT Expression vector (ThermoFisher) with an N-terminal 3 × FLAG tag for transient expression in 293T and RPE1 cells and for stable expression in FlpIn T-REx 293 cells. For recombinant expression in *E. coli*, SAF-A derivatives were cloned into a pET32a vector with an N-terminal 6 × His tag. For PLA, T7 SAF-A derivatives were cloned into a modified pCGN vector where the HA tag was replaced by a T7 tag.

#### RNA interference

For siRNA treatment, cells (10%–20% confluent) were transfected with 10 nM Stealth RNA (ThermoFisher) using Lipofectamine RNAi MAX (ThermoFisher) for the time indicated. Stealth RNA sequences and the identification numbers are CCUGGGAAUCGTGGCGGATATAATA HSS104917 for SAF-A and GCUUUGCCUUCGUUCAGUAUGUUAA HSS179304 for hnRNP C. The control RNA is Stealth RNAi siRNA Negative Control, Med GC (ThermoFisher).

For shRNA treatment, RPE1 cells (80% confluent) were transfected with pSuper-puro vector harboring hnRNP A1 target sequence using Lipofectamine 2000 (ThermoFisher). After 48 hr selection with 3 μg.ml^-1^ puromycin, DNA-FISH and western blotting were performed.

#### SAF-A extraction

To analyze Triton X-100 soluble/insoluble SAF-A, cells were treated with CSK buffer containing 100 mM NaCl, 0.1% Triton X-100, 300 mM Sucrose, 1 mM MgCl_2_, 1 mM EGTA, 10 mM PIPES (pH 6.8), and 100 μM PMSF (phenylmethylsulfonyl fluoride) on ice for 10 min, followed by immuno-staining or divided into the supernatant and pellet fractions by centrifugation, at 5000 rpm, 4°C for 5 min, for western blotting. To analyze DNase or RNase A/T1 extraction of SAF-A, cells were treated with CSK buffer containing 100 units.mL^-1^ DNase I (ThermoFisher) and 2.5 mM CaCl_2_ or 0.5/20 units.ml^-1^ RNase A/T1 (Ambion) for 30 min at R.T. and were divided into the supernatant and pellet by centrifugation, at 5000 rpm, 4°C for 5 min.

#### Oligomerization assay

1 × 10^6^ 293T cells were exposed to disuccinimidyl suberate (DSS; 1 mM, 0.3 mM, 0.1 mM), 1,8-bismaleimido-diethyleneglycol (BM(PEG)2; 1 mM, 0.3 mM, 0.1 mM) or N-γ-maleimidobutyryl-oxysuccinimide ester (GMBS; 1 mM, 0.3 mM, 0.1 mM) crosslinking for 5 min at R.T. in PBS or permeabilised with 100 μL of reaction buffer including 125 mM NaCl, 50 mM Tris pH 7.0 (for BM(PEG)2) or 50 mM PIPES pH7.0 (for DSS or GMBS), 5 mM MgCl_2_ and 0.1% Triton X-100 at 37°C for 10 min before crosslinking. After quenching DSS with 20 mM Tris pH 7.0, BM(PEG)2 with 20 mM L-cysteine (Sigma-Aldrich) or GMBS with 20 mM Tris pH 7.0 and 20 mM L-cysteine, cells were processed for western blotting. 0.3 mM cross-linker was used for routine experiments while 1 mM was used for SAF-A IPs (Figures 4C and 4E).

For Figure 4B right, reaction buffer was supplemented with 5 mM ATP or ATPγS. For Figure 4D, after pre-treatment with 3 μg.ml^-1^ PureLink RNaseA (ThermoFisher) in reaction buffer at 37°C for 10 min, 293T cell lysates were incubated in the presence or absence of 30 μg.ml^-1^ total RNA from 293T cells and 5 units.ml^-1^ Apyrase (NEB) at 37°C for 10 min, following by crosslinking. After RNaseA treatment reaction buffer was supplemented with RNasin Plus RNase Inhibitor (Promega) 1000 units.ml^-1^, 1 mM DTT and 5 mM CaCl_2_.

#### Protein gels and western blotting

Cells were suspended in NuPAGE LDS sample buffer (ThermoFisher) with 10 mM DTT, incubated at 100°C for 5 min and sonicated briefly. For SAF-A oligomer blots, protein samples were resolved on 5 or 8% bis-tris gels or 7% tris-acetate gels (ThermoFisher) and transferred to Immobilon-P PVDF 0.45 μm membrane (Merck Millipore) by wet transfer. Membranes were probed with antibodies using standard techniques and detected by enhanced chemiluminescence.

#### Immunoprecipitation and Native PAGE

FLAG-tagged SAF-A expressed in 293T cells was cross-linked with 1 mM 1,8-bismaleimido-diethyleneglycol (BM(PEG)2) or 1 mM dithio-bis-maleimidoethane (DTME), was extracted by sonication (10 × 30 s at amplitude 2 μm), immuno-purified using anti-FLAG M2 agarose beads (Sigma-Aldrich) and eluted with 100 μg.ml^-1^ 3 × FLAG peptide (Sigma-Aldrich) suspended in 50 mM NaCl, 50 mM Tris pH 7.0, 5 mM MgCl_2_ and 0.1% Triton X-100. For Figure 4E, immunoprecipitants were incubated in the presence or absence of 10 mM DTT to reverse crosslinks, 0.5/20 units.ml^-1^ RNase A/T1, 5 units.ml^-1^ Apyrase, 1 mM ATP or 1mM ATPγS for 30 min at 37°C. A 4%–16% NativePAGE bis-tris gel (ThermoFisher) was used for native protein fractionation, according to the manufacturer’s instructions. In Figure 4C, 5-ethynyl uridine was conjugated to 0.2 mM biotin azide (PEG4 carboxamide-6-azidohexanyl biotin; ThermoFisher) using click chemistry, transferred to PVDF membrane, followed by incubation with ExtrAvidin−Peroxidase (Sigma-Aldrich) and detected with SuperSignal West Femto (ThermoFisher).

#### Purification of recombinant proteins

To obtain SAF-A proteins, E.coli BL21 (DE3) cells transformed with a pET32a vector encoding SAF-A cDNA were grown in 2 × YT medium (16 g.ml^-1^ Tryptone, 10 g.ml^-1^ Yeast extract, 5 g.ml^-1^ NaCl) supplemented with 20 μg.ml^-1^ ampicillin and 20 μg.ml^-1^ chloramphenicol. Protein expression was induced overnight at 18°C with 1 mM IPTG after OD600 reached 0.6. Cells were lysed in buffer containing 50 mM Tris-HCl (pH 8.0), 500 mM NaCl, 60 mM imidazole, 10% glycerol, 0.1% Triton X-100 and 1 mM DTT. The SAF-A proteins were affinity-purified by HisTrap HP (GE Healthcare Life Sciences), dialysed into 150 mM NaCl, 30 mM imidazole (pH 7.0) and 1 mM DTT overnight at 4°C and were further purified by CM-Sepharose cation exchange chromatography (Sigma-Aldrich) and eluted using a 150 to 500 mM NaCl gradient in dialysis buffer.

#### ATPase activity assay

An ATPase activity assay was performed with 3.0 μm SAF-A protein in reaction buffer (50 mM Tris–HCl (pH 7.5), 125 mM NaCl, 10 mM MgCl_2_, 5 mM DTT, 1 mM ATP or 1 mM GTP) in a final volume of 50 μL for 30 min at 37°C. The 10 μL reaction was quenched with 90 μL of BIOMOL Green (Enzo Life Science) and free phosphate was detected by absorbance at 620 nm using a Tecan M200Pro. A phosphate standard curve was used to estimate the amount of phosphate released during ATP hydrolysis. Error bars denote standard deviations from three independent samples.

#### Fluorescence studies by luminescence spectroscopy

Fluorescence experiments were performed at 37°C using a LS 50 PerkinElmer luminescence spectrometer. Reactions comprised 4 μm SAF-A in 50 mM Tris–HCl (pH 7.5), 125 mM NaCl, 10 mM MgCl_2_, 5 mM DTT. Spectra for SAF-A AAA+ fragments were obtained with 290 nm excitation wavelength and fluorescence changes calculated at 360 nm. All fluorescence measurements were performed in duplicate.

#### Proximity ligation assay (PLA)

FlpIn T-REx 293 cells carrying an inducible copy of wild-type or mutant SAF-A were transfected with stealth RNAi to knock down endogenous SAF-A using RNAi MAX (ThermoFisher) for 24 hr. Cells were then transfected with plasmids encoding T7-tagged SAF-A or point mutants with Lipofectamine 3000 (ThermoFisher) and the expression of FLAG-tagged-SAF-A was induced with doxycycline at the same time. After 24 hr, PLA was performed according to the manufacturer’s instructions using Duolink in situ (Sigma-Aldrich).

#### [5-3H]uridine incorporation

Nascent transcription was measured as described (Naughton et al., 2013).

#### 5-EU incorporation

Cells were treated with 1 mM 5-ethynyl uridine (Base Click) and 1 mM thymidine (Sigma-Aldrich) for the indicated time. In Figure S6C, cells were pretreated with 50 ng.ml^-1^ actinomycin D. Click reaction was performed with Click-iT EdU Alexa Fluor 647 Imaging Kit or Click-iT Nascent RNA Capture Kit, according to the manufacturer’s instructions (ThermoFisher).

#### 4-thiouridine incorporation and quantification of nucleosides

After 30 min incubation with 0.5 mM 4-thiouridine cells were rinsed with PBS and lysed in DNA lysis buffer (100 mM EDTA, 20 mM Tris pH 7.5, 50 mM NaCl 0.5% SDS and 300 μg.ml^-1^ protease K). The cell lysate was incubate at 37°C for 30 min and precipitated in 2 volumes of ethanol. The precipitate was suspended in 70% ethanol overnight and resuspended in 50 μL of combined DNA and RNA lysis buffer (1 × DNaseI digestion buffer (NEB) with 1 mM ZnSO4). The nucleic acid was digested with 1 μL Dnase I (NEB) and 2 μL (1 mg.ml^-1^) nuclease P1 for 24 hr at 37°C. After this the sample was denatured by heat (95°C for 5 min) and rapidly cooled on ice. 2 volumes of 30 mM Na acetate pH 5.2, (1 mM ZnSO4) was added and the sample was digested with fresh DNaseI (1 μl) and Nuclease P1 (2 μl) for a further 24 hr at 37°C. 50 μL of lysate was separated by reverse phase HPLC.

HPLC was carried out on a Dionex Ultimate 3000 HPLC system equipped with Chromeleon software, a column chiller (8°C), 5 μm APEX ODS C18 column, 50 mM ammonium phosphate (monobasic) mobile phase (1 ml.min^-1^), and a UV absorbance detector capable of collecting UV absorbance at 5 wavelengths simultaneously. Nucleotides were detected at their peak absorbances: thymidine 5′monophosphate (in DNA) at 268 nm (retention time 22.5 min), guanosine monophosphate (in RNA) at 254 nm (retention time 11.5 min), and 4-thiouridine monophosphate (in RNA) at 323 nm (retention time 14.7 min). Nucleotide quantities were measured by the areas under each peak at the respective peak UV absorbances.

#### RNA Sequencing and analysis

Total RNA from cells transfected with siRNA for the time indicated were prepared with RNeasy Mini kit (QIAGEN) and treated with ribo-Zero kit (illumina) to remove ribosomal RNA. RNA-seq libraries were prepared with a NEBNext® Ultra RNA Library Prep Kit for Illumina (NEB), according to manufacturer’s instructions, and were quantified using a BioAnalyzer 2100 with a High Sensitivity DNA Kit (Agilent). RNA-seq Libraries were analyzed by Edinburgh Genomics at the University of Edinburgh, UK, for quality control and sequencing (HiSeq2500, 50 base single-end reads). RNA-seq data were processed using a standard TopHat and Cufflinks methodology (Trapnell et al., 2012). Single end reads were aligned using Tophat 2.0.10 and Bowtie 2.1.0 using a human hg19 bowtie2 index. Aligned reads were processed using Cufflinks 2.2.1 with a human hg19 reference annotation and differential gene expression calculated between control and knockdown samples with Cuffdiff using a 0.0005 p value cut off.

#### Three-dimensional RNA/DNA fluorescence in situ hybridization

3D RNA/DNA FISH was performed as described (Naughton et al., 2010). Fosmid probes (BacPac resources), human LINE1 cDNA and human C0t1 DNA (ThermoFisher) were labeled in digoxigenin-11-UTP or biotin-16-dUTP. Custom MyTags libraries for Oligo-DNA-FISH were synthesized to cover 20 kb of genomic sequence positioned every 1 Mb across a 10 Mb region of the genome (MYcroarray). Gene rich library probes labeled with ATTO 488 cover chr11: 760000 – 10980000. Gene poor library probes labeled with ATTO 550 cover chr11: 20000000 – 29200000. Each 5 μm probe was hybridized as for DNA-FISH. Chromosome Painting was performed using a chromosome 2 painting probe labeled with Texas Red and a chromosome 4 painting probe labeled with FITC (Cytocell) or a chromosome 18 or chromosome 19 paint (MetaSystem) directly labeled with Spectrum Orange or FITC, respectively.

#### Image capture and analysis

Image capture and analysis was performed as described (Naughton et al., 2013) and image color balance was adjusted to improve data visualization in the manuscript.

#### STED microscopy

Super-resolution images were acquired using a STED microscope. Flp-In T-REx 293 cells depleted of endogenous SAF-A (48 h) and then doxycycline induced for 24 hr to express wild-type or mutant SAF-A were prepared on high precision cover-glass (Zeiss, Germany). STED images were acquired on a Leica SP5 SMD g-STED microscope (Leica Microsystems, Germany) using a 100 × objective lens (HC PL APO 100 × /1.40 Oil STED White) with immersion oil (Leica Type F, refractive index 1.5180). A confocal image was taken using a 488 nm laser for FLAG-SAF-A and 647 nm laser for DRAQ7 stained DNA. The STED image was taken using 488 nm for excitation from a white light, super continuum laser and a pulsed 592 nm laser for depletion. Detection was with a HyD detector with a time gate of 0.5-6 ns. STED image processing and analysis were carried out using the STED module of the LAS AF (Leica Application Suite Advanced Fluorescence). STED resolution was assessed by imaging 20 nm fluorescent beads (ThermoFisher). To measure image granularity nuclei were segmented on DRAQ7 to exclude background and nucleoli using an iVision script. The resulting mask was used to isolate SAF-A image data, background signal was removed and granules were segmented based on intensity script and quantified for each nucleus.

#### Fluorescence lifetime imaging microscopy (FLIM) data acquisition

Förster Resonance Energy Transfer (FRET) is a process whereby the transfer of energy occurs from an excited state fluorophore (donor) to a second chromophore (acceptor) in close proximity and is used to detect molecular interactions in vivo where two molecules are less than 10 nm apart. In FLIM-FRET microscopy, fluorescence lifetime is measured as a quantitative readout of FRET, with interactions causing a decrease in the fluorescence lifetime of the donor molecules. We performed FLIM-FRET experiments with 293T cells transiently transfected with either GFP-AAA+/RGG SAF-A fragment (donor alone) or GFP-AAA+/RGG and mCherry-AAA+/RGG together to investigate the oligomerization of SAF-A in different conditions. To deplete endogenous SAF-A, 293T cells were transfected with stealth RNA using RNAiMAX for 24 hr and transfected with GFP-AAA/+RGG and mCherry-AAA+/RGG wild-type or point mutants with Lipofectamine 3000 for 24 hr. Acquisition was performed on a Leica SP5 SMD (Single Molecule Detection) confocal laser-scanning microscope using a 63 × 1.4NA HCX PL Apo oil immersion objective lens. The GFP donor fluorophore was excited using a tunable white light laser operating at 488 nm and 40 MHz pulse rate. Emission was detected with an external single photon avalanche diode (MicroPhoton Devices) and photon arrival time relative to the laser pulse was determined using a PicoHarp 300 time-correlated single photon counting (TCSPC) module. FLIM measurements were integrated for 120 s with a 512 × 512 pixel format at a maximum photon count rate of 105 counts per second and subsequently binned to a 256 × 256 pixel format to maximize photon counts per pixel for accurate data fitting.

#### FLIM-FRET analysis

FLIM analysis was carried out using SymPhoTime v5.4.4 (PicoQuant). The measured fluorescence decay was fitted to a bi-exponential decay model using the maximum-likelihood estimation method. Fluorescence lifetime values were determined by pixel-by-pixel fitting, which was guided by an initial global decay fit. Reported fluorescence lifetimes are an amplitude-weighted average of the two decay components, т_amp_ *=* (A_1_т_1_ + A_2_т_2_) / (A_1_ + A_2_) (where A_1_ and A_2_ are the amplitudes of the decay components and т_1_ and т_2_ are the fluorescence lifetimes) and were only calculated for pixels with greater than 300 photon counts. FRET efficiency, E_FRET_ = 1 – т_DA_/т_D_, where τ_DA_ is the lifetime of the donor (GFP) in the presence of the acceptor (mCherry) and т_D_ is the single pixel median amplitude-weighted fluorescence lifetime reported for the GFP donor-only sample, calculated per pixel from the GFP-mCherry samples. Image maps representing FRET efficiency per pixel were constructed in R using the spatstat package.

#### Protein sequence analysis and domain identification

The domain composition of SAF-A was initially observed using SMART (Schultz et al., 1998) and then the most likely domain sequence lengths extended on the basis of homology searches of the Protein Data Bank (PDB) taking into consideration additional conserved secondary structure elements for the SAP (PDB ID: 1ZRJ), SPRY (PDB ID: 3TOJ) and AAA (3ZVL) domains, respectively. The C-terminal RNA binding region was annotated on the basis of functional studies (Kiledjian and Dreyfuss, 1992). The disordered regions were predicted using MetaPrDOS (Ishida and Kinoshita, 2008) by integrating results from 5 disorder prediction methods (see Figure S3A).

#### Fold recognition and 3D homology modeling of SAF-A AAA domain

The SAF-A isoform 2 AAA domain sequence (aa 469-653) was analyzed using PHYRE-2 (Kelley et al., 2015) to assess fold compatibility. All returned top hits were AAA superfamily related with 100% confidence. The AAA domain from the crystal structure of the mammalian polynucleotide kinase 3′ phosphatase PDB ID: 3ZVL chain A (1.65 Å) was selected as a template for modeling the SAF-A AAA domain based upon an HHPred search of the PDB70 database. The HMM-HMM target-template alignment was used as input for modeling after manual checking of alignment, secondary structure equivalence, gap positioning and undertaking minor editing. A PsiPred secondary structure prediction identified an additional predicted α -helix (aa 512-521) and an extended α-helix (aa 533-554) when compared with the template located after the canonical β strand 2; these additional predicted secondary structure features were restrained during the model building process. A total of 100 models were built using Modeler 9v12 (Eswar et al., 2007) and the model with the lowest “DOPE” energy (Shen and Sali, 2006) was selected as the representative model, and assessed for valid stereochemistry (Ramachandran plot: 98.9% of residues in favored and allowed regions) and packing quality (average Z-score −1.04). PyMol (http://www.pymol.org) was used for 3D visualization, analysis and figure preparation. Pro-origami was used to create the 2-D cartoon topology schematic (Stivala et al., 2011). The target-template alignment was generated and annotated using EsPript v3 (Gouet et al., 1999).

### Quantification and Statistical Analysis

The statistical significance of compaction was tested using a nonparametric Mann–Whitney U (Wilcoxon) test (using R programming). p < 0.05 was taken as statistically significant. For oligo probe DNA-FISH analysis, the Woolz image processing system, initially developed for the eMouseAtlas program (Armit et al., 2015), was used to analyze the distribution of 3D DNA-FISH spot images. MAPaint, a Woolz based interactive 3D segmentation tool, was used to delineate spot domains. From these domains convex hulls and their volumes were then computed. The open source Woolz image processing system is freely available from https://github.com/ma-tech/Woolz. For analyzing DAPI texture cells were grown overnight on slides, stained with DAPI and mounted. 12 bit images were collected using a 405 nm laser on a SP5 confocal microscope (Leica) using a 100 × objective and were segmented to exclude background and nucleoli using a custom iVision (BioVis) script. Segmented nuclei were sub-sampled 100 × each with an 8 × 8, 12 × 12, 16 × 16, 20 × 20, 24 × 24 and 28 × 28 window (i.e., 600 measurements per nucleus using an iVision script). Sub-sampled images were imported into R and visualized using the spatstat package. To quantify texture images were transformed to a gray level co-occurrence matrix (GLCM) using the radiomics package and second order matrix statistics were calculated.

### Data and Software Availability

The accession number for RNA sequencing reported in this paper is NCBI GEO: GSE98541.

## Author Contributions

R.-S.N., L.B., C.N., A.R.D., M.A., B.R., P.C.B., S.K.M., and N.G. performed laboratory experiments. D.C.S., A.B., B.H., R.S.S., and B.R. undertook data analysis. R.-S.N., L.B., R.R.D., S.K.M., and N.G. designed the study. R.-S.N., L.B., and N.G. wrote the manuscript with contributions from all the authors.
