# Supplementary material for: SAF-A Regulates Interphase Chromosome Structure through Oligomerization with Chromatin-Associated RNAs
Source: Cell. 2017 Jun 15;169(7):1214–1227.e18. doi: 10.1016/j.cell.2017.05.029 (PMC5473940; doi:10.1016/j.cell.2017.05.029)
Supplement: Document S1. Table S1 [file mmc1.pdf]

Cell, Volume *169*

Supplemental Information

SAF-A Regulates Interphase Chromosome Structure through Oligomerization with Chromatin-Associated RNAs

Ryu-Suke Nozawa, Lora Boteva, Dinesh C. Soares, Catherine Naughton, Alison R. Dun, Adam Buckle, Bernard Ramsahoye, Peter C. Bruton, Rebecca S. Saleeb, Maria Arnedo, Bill Hill, Rory R. Duncan, Sutherland K. Maciver, and Nick Gilbert

Table S1. List of fosmids, Related to Figures 1, S1, 6, S6.

Position	Clone names	Sequence name	Co-ordinates (hg38)	Properties
11p15.5	CI 11p15-49		chr11:736369	gene rich
11p15.5	H19-IGF2		chr11:2070000	gene rich
11p15.1	WI2-2224K21	G248P87004F11	chr11:17,488,939-17,528,052	gene rich
11p15.1	WI2-3168N23	G248P8017G12	chr11:19,034,283-19,072,361	gene rich
11p14.1	WI2-906E12	G248P8190C6	chr11:29,684,660-29,731,106	gene poor
11p14.1	WI2-1673H3	G248P83109D2	chr11:31,195,720-31,235,826	gene poor
Xq13.1	WI2-2977E1	G248P89772C1	chrX:69862989-69904969	gene rich
Xq13.1	WI2-1660A18	G248P87866A9	chrX:71,851,120-71,887,633	gene rich
Xq24	WI2-2726C8	G248P88902B4	chrX:121,055,336-121,094,297	gene poor
Xq25	WI2-465K3	G248P8676F2	chrX:123,014,421-123,054,105	gene poor
1p31.2	WI2-2013D5	G248P86197B3	chr1:68350693-68390943	gene poor
1p31.2	WI2-2446E1	G248P83504C1	chr1:69,563,715-69,605,049	gene poor
2p25.1	WI2-1914D7	G248P86693B4	chr2:11,338,760-11,377,104	gene rich
2p25.1	WI2-2004M11	G248P85784G6	chr2:11,800,555-11,838,935	gene rich
21q22.3	WI2-2110F20	G248P86155C10	chr21:42,316,026-42,353,544	gene rich
21q22.3	WI2-2736C8	G248P88986B4	chr21:42,398,031-42,434,239	gene rich

Fosmids were obtained from BacPac resources, DNA co-ordinates are hg38 and gene density was calculated from Ensembl 87.
